# A synthetic peptide that prevents cAMP regulation in mammalian hyperpolarization-activated cyclic nucleotide-gated (HCN) channels

**DOI:** 10.7554/eLife.35753

**Published:** 2018-06-20

**Authors:** Andrea Saponaro, Francesca Cantini, Alessandro Porro, Annalisa Bucchi, Dario DiFrancesco, Vincenzo Maione, Chiara Donadoni, Bianca Introini, Pietro Mesirca, Matteo E Mangoni, Gerhard Thiel, Lucia Banci, Bina Santoro, Anna Moroni

**Affiliations:** 1 Department of BiosciencesUniversity of MilanMilanItaly; 2 Department of ChemistryUniversity of FlorenceFlorenceItaly; 3 Magnetic Resonance CenterUniversity of FlorenceFlorenceItaly; 4 Interuniversity Consortium for Magnetic Resonance of MetalloproteinsSesto FiorentinoItaly; 5 Institut de Génomique FonctionnelleCNRS, INSERM F-34094, Université de MontpellierMontpellierFrance; 6 Laboratory of Excellence Ion Channels Science and TherapeuticsValbonneFrance; 7 Department of BiologyTU-DarmstadtDarmstadtGermany; 8 Institute of Neurosciences, Consiglio Nazionale delle Ricerche, Florence, Italy; 9 Department of NeuroscienceColumbia UniversityNew YorkUnited States; 10 Institute of BiophysicsConsiglio Nazionale delle RicercheMilanItaly; J.W. Goethe-UniversityGermany

**Keywords:** cardiac pacemaker, HCN, cAMP, Mouse

## Abstract

Binding of TRIP8b to the cyclic nucleotide binding domain (CNBD) of mammalian hyperpolarization-activated cyclic nucleotide-gated (HCN) channels prevents their regulation by cAMP. Since TRIP8b is expressed exclusively in the brain, we envisage that it can be used for orthogonal control of HCN channels beyond the central nervous system. To this end, we have identified by rational design a 40-aa long peptide (TRIP8b_nano_) that recapitulates affinity and gating effects of TRIP8b in HCN isoforms (hHCN1, mHCN2, rbHCN4) and in the cardiac current I_f_ in rabbit and mouse sinoatrial node cardiomyocytes. Guided by an NMR-derived structural model that identifies the key molecular interactions between TRIP8b_nano_ and the HCN CNBD, we further designed a cell-penetrating peptide (TAT-TRIP8b_nano_) which successfully prevented β-adrenergic activation of mouse I_f_ leaving the stimulation of the L-type calcium current (I_CaL_) unaffected. TRIP8b_nano_ represents a novel approach to selectively control HCN activation, which yields the promise of a more targeted pharmacology compared to pore blockers.

## Introduction

Hyperpolarization-activated cyclic nucleotide-gated (HCN1-4) channels are the molecular correlate of the I_f_/I_h_ current, which plays a key role in controlling several higher order physiological functions, including dendritic integration and intrinsic rhythmicity both in cardiac and neuronal cells (Robinson and Siegelbaum, 2003). Unique among the voltage-gated ion channel superfamily, HCN channels are modulated by the direct binding of cAMP to their cyclic nucleotide binding domain (CNBD). Binding of the cyclic nucleotide increases the channel open probability upon hyperpolarization via conformational changes in the CNBD that are propagated to the pore through the C-linker domain (DiFrancesco and Tortora, 1991; Wainger et al., 2001; Zagotta et al., 2003).

In addition to cAMP, HCN channels are regulated by TRIP8b, a brain-specific auxiliary (β) subunit, which modulates two independent features of the channel, namely trafficking and gating (Santoro et al., 2009; Zolles et al., 2009). For this dual regulation, TRIP8b binds HCN channels through two distinct sites: via the tetratricopeptide repeat (TPR) domain, which interacts with the last three amino acids (SNL) of HCN channels and regulates their trafficking; and via the TRIP8b_core_ domain, which interacts with the CNBD and antagonizes the effect of cAMP on the voltage dependency of the channel (Santoro et al., 2011; Han et al., 2011; Hu et al., 2013).

Here, we focus our attention on the specific action of TRIP8b in preventing cAMP regulation of HCN channels. Given the brain-specific localization of TRIP8b, we posit that a TRIP8b-derived peptide drug, able to reproduce the effect of the full length protein on HCN channel gating, can be developed for orthogonal selective regulation of HCN in cells/tissues in which TRIP8b is not expressed. cAMP-dependent modulation of HCN channels underlies distinct roles of cAMP in heart rate regulation (DiFrancesco, 1993) and development of peripheral neuropathic pain (Emery et al., 2012; Herrmann et al., 2017), which can be dissected by using a TRIP8b-based tool. In this regard, peptide-based drugs (2–50 aa long) are emerging as a fascinating application area as they open new therapeutic possibilities with an advantage over small molecules in terms of specificity and affinity for the target (Fosgerau and Hoffmann, 2015; Henninot et al., 2018). To this end, we searched for the minimal peptide that binds to the CNBD and recapitulates the gating effect of full length TRIP8b in three HCN isoforms (HCN1, HCN2 and HCN4) and in the native I_f_ current. In previous studies, we identified the core portion of TRIP8b (TRIP8b_core,_ 80 aa long) that interacts with the HCN CNBD and prevents cAMP modulation in full length channels (Santoro et al., 2011; Hu et al., 2013; Saponaro et al., 2014). A recent paper (Lyman et al., 2017) reported an even shorter binding sequence of TRIP8b (37 aa). However, this peptide, which was identified by progressive truncation of TRIP8b_core_, failed to reproduce the binding affinity of the starting construct. Moreover, evidence for activity of this peptide on HCN currents is lacking. In the present study, we adopted a structure-driven rational design approach to engineer a 40-aa long peptide, TRIP8b_nano_, that efficiently prevents cAMP regulation of HCN channels. The rational design of this peptide, based on secondary structure predictions and on NMR data of TRIP8b_core_, was supported by an NMR-based 3D model structure of the complex formed by the TRIP8b_nano_ peptide and CNBD of the human HCN2 channel isoform. This structural information identifies crucial interactions between the two partners and explains both direct (Han et al., 2011; DeBerg et al., 2015; Bankston et al., 2017) and indirect (allosteric) (Hu et al., 2013; Saponaro et al., 2014) modes of competition between TRIP8b and cAMP for binding to the CNBD. The evidence that TRIP8b_nano_ establishes all relevant interactions with the CNBD is reflected by the finding that, contrary to shorter core sequences (Lyman et al., 2017), it binds to the isolated CNBD with the same affinity as TRIP8b_core_ and acts with even higher efficacy than TRIP8b_core_ in preventing cAMP modulation of full length HCN channels (Hu et al., 2013). In pacemaker myocytes of the sino-atrial node (SAN), TRIP8b_nano_ equally prevented cAMP stimulation of native f-channels leading to a 30% reduction in spontaneus firing rate.

To develop TRIP8b_nano_ as a membrane permeable drug, we linked it with the positively charged TAT sequence (Herce et al., 2014). TAT-TRIP8b_nano_ was tested in SAN pacemaker myocytes where its addition to the extracellular buffer prevented adrenergic stimulation of the I_f_ current leaving the activation of the L-type calcium current (I_CaL_) unaffected. Our study opens the possibility of selective in vivo control of the cAMP-dependent facilitation of HCN channel opening, by local supply of TAT-TRIP8b_nano_ peptide.

## Results

We have previously shown that TRIP8b_core_ (residues 223–303 of mouse TRIP8b splice variant 1a4, hereafter TRIP8b) interacts with two elements of the isolated CNBD protein fragment from HCN channels (residues 521–672 of human HCN2, hereafter CNBD): the C-helix and the N-bundle loop, a sequence connecting helix E’ of the C-linker with helix A of the CNBD (Saponaro et al., 2014). Biochemical assays confirmed that each of these two elements, that is, the N-bundle loop and C-helix, is necessary but not sufficient for binding (Saponaro et al., 2014).

**Figure 1.**
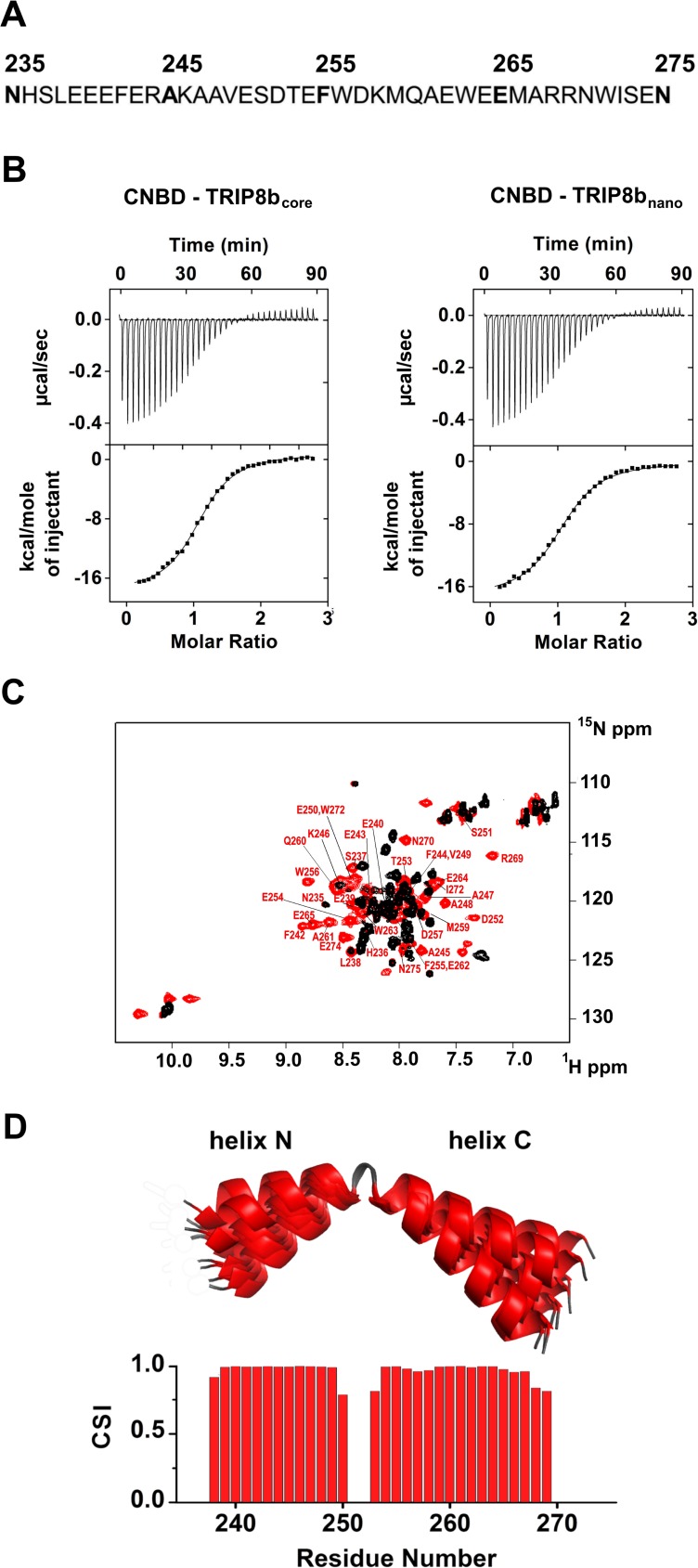
Functional and structural characterization of TRIP8b_nano_. (**A**) Primary sequence of TRIP8b_nano_. Amino acid numbering refers to full length mouse TRIP8b (1a4). (**B**) Binding of TRIP8b_core_ and TRIP8b_nano_ to purified His_6_-MBP-CNBD measured by Isothermal titration calorimetry (ITC). Upper panel, heat changes (μcal/sec) during successive injections of 8 μL of the corresponding TRIP8b peptide (200 μM) into the chamber containing His_6_-MBP-CNBD (20 μM). Lower panel, binding curve obtained from data displayed in the upper panel. The peaks were integrated, normalized to TRIP8b peptide concentration, and plotted against the molar ratio (TRIP8b peptide/His_6_-MBP-CNBD). Solid line represents a nonlinear least-squares fit to a single-site binding model, yielding, in the present examples, a K_D_ = 1.2 ± 0.1 μM for TRIP8b_core_ and K_D_ = 1.4 ± 0.1 μM for TRIP8b_nano_. (**C**) Evidence for TRIP8b_nano_ folding upon CNBD binding based on the superimposition of the [^1^H, ^15^N] heteronuclear single quantum coherence (HSQC) NMR spectrum of CNBD-free TRIP8b_nano_ (black) and CNBD-bound TRIP8b_nano_ (red). The latter experiment was performed at the molar ratio ([CNBD]/[TRIP8b_nano_])=3. The backbone amide (HN) signals of the residues of CNBD-bound TRIP8b_nano_ are labelled in red. (**D**) (Top) Ribbon representation of the 10 lowest energy conformers of TRIP8b_nano_ bound to CNBD used for in silico modelling of CNBD-TRIP8b_nano_ complex. The unfolded regions at the N- and C-termini of the construct (residues 235–237 and 270–275) are omitted for clarity. (Bottom) Chemical Shift Index (CSI, calculated using TALOS+) plotted as a function of the residue number of TRIP8b_nano_ bound to CNBD. Positive values represent helical propensity.

To understand the interaction in atomic detail, we used solution NMR spectroscopy to characterize the structural properties of the CNBD - TRIP8b_core_ complex. However, the NMR spectra of TRIP8b_core_ showed very few signals. In order to improve the quality of the NMR spectra, we reduced the length of the TRIP8b fragment by progressively removing residues at the N- and C-termini with no predicted secondary structure. The truncated peptides were then tested for CNBD binding activity by isothermal titration calorimetry (ITC). We thus identified a 40-aa peptide (TRIP8b_nano_, comprising residues 235–275 of TRIP8b, Figure 1A) with a binding K_D_ of 1.5 ± 0.1 µM, a value similar to the K_D_ of 1.2 ± 0.1 µM obtained with TRIP8b_core_ (Figure 1B). TRIP8b_nano_ was therefore employed for all subsequent NMR experiments, resulting in a remarkable improvement in the spectral quality and sample stability.

### Structural characterization of TRIP8b_nano_ bound to CNBD

The comparison of the ^1^H-^15^N HSQC spectra of TRIP8b_nano_ with and without CNBD bound shows that the peptide folds upon interaction with the CNBD. Thus, the ^1^H-^15^N HSQC spectrum of TRIP8b_nano_ without CNBD shows a limited ^1^H resonance dispersion, characteristic of unstructured proteins (Dyson and Wright, 2004), while a larger number of well-dispersed amide signals appear in the spectrum of the CNBD-bound form (Figure 1C). Importantly, we were now able to assign the backbone chemical shift resonances of TRIP8b_nano_ bound to the CNBD. The φ and ψ dihedral angles obtained from the NMR assignment indicate that the peptide displays two α-helices (stretch L_238_-E_250_ named helix N and stretch T_253_-R_269_ named helix C) when bound to CNBD. The helices are separated by two amino acids; three and six residues at the N- and C- termini, respectively, are unstructured (Figure 1D).

### Structural characterization of CNBD bound to TRIP8b_nano_

**Figure 2.**
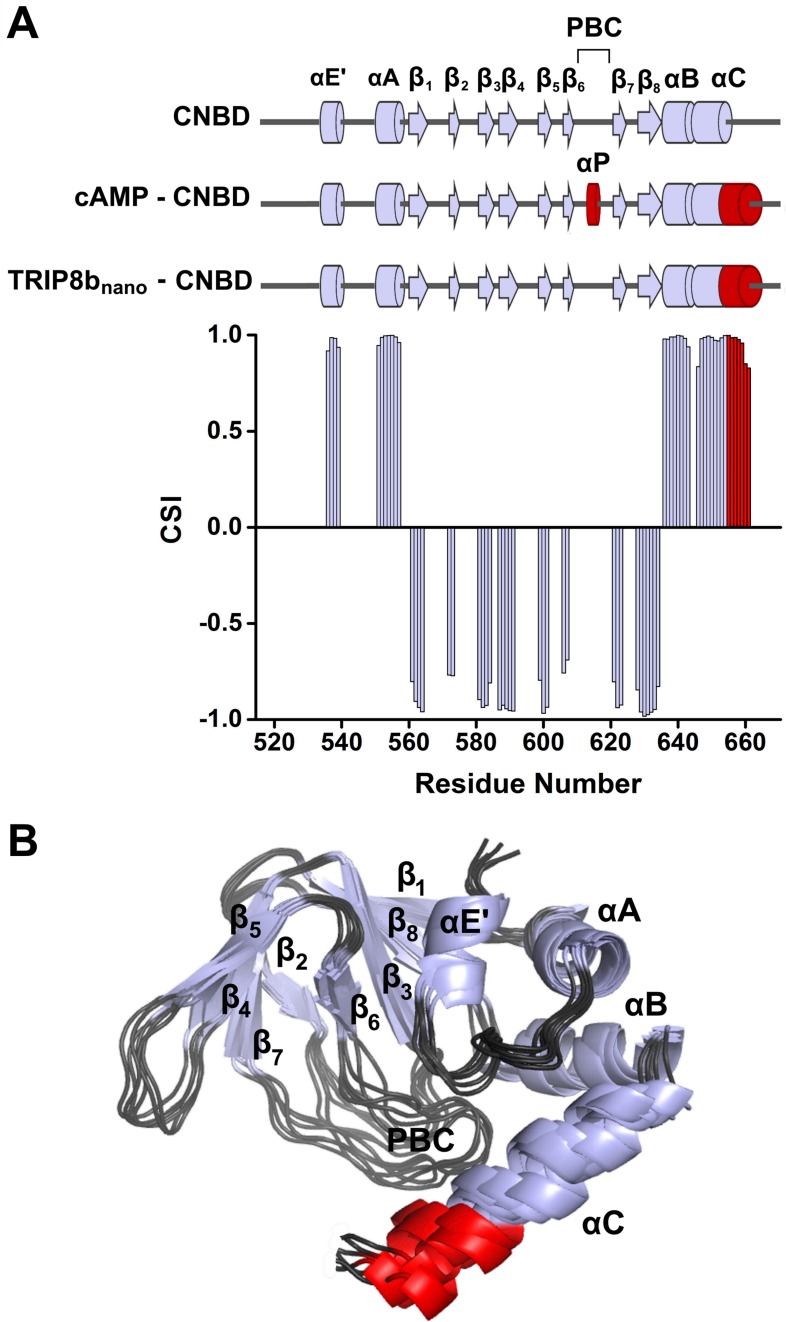
NMR structure of CNBD bound to TRIP8b_nano_. (**A**) (Top) comparison of secondary structure elements of cAMP-free CNBD (Saponaro et al., 2014), cAMP-bound CNBD (Zagotta et al., 2003) and cAMP-free CNBD bound to TRIP8b_nano_ (this study). Secondary structure elements are indicated by arrows (β-strands) and cylinders (α-helices) and labeled. The loop between β_6_ and β_7_ constitutes the Phosphate Binding Cassette (PBC). The elements that fold upon binding of cAMP and TRIP8b_nano_ are shown in red. (Bottom) Chemical Shift Index (CSI, calculated using TALOS+) plotted as a function of the residue number of CNBD bound to TRIP8b_nano_. Positive values represent helical propensity, while negative values represent strands. (**B**) Ribbon representation of the 10 lowest energy conformers of CNBD bound to TRIP8b_nano_ used for in silico modeling of CNBD - TRIP8b_nano_ complex. Secondary structure elements are coloured in light gray and labeled. Loop regions are colored in dark gray. The distal region of the C-helix (residues 657–662), which is unfolded in the free form of the CNBD (Saponaro et al., 2014) and folds upon TRIP8b_nano_ binding, is coloured in red. The unfolded regions at the N- and C-termini of the construct (residues 521–532 and 663–672 respectively) are omitted for clarity.

NMR-analysis of the CNBD fragment bound to TRIP8b_nano_ revealed that the interaction with the peptide does not affect the overall fold of the protein. Thus, the CNBD adopts the typical fold of the cAMP-free state, in line with previous evidence that this is the form bound by TRIP8b (Saponaro et al., 2014; DeBerg et al., 2015) More specifically, the secondary structure elements of the cAMP-free CNBD are all conserved in the TRIP8b_nano_–bound CNBD (Figure 2). This finding generally agrees with a previous double electron-electron resonance (DEER) analysis of the CNBD - TRIP8b interaction, which showed that TRIP8b binds to a conformation largely similar to the cAMP-free state (DeBerg et al., 2015). Despite the overall agreement with the DEER study, the NMR data also reveal a new and unexpected feature of TRIP8b binding to the CNBD. Indeed, our results show that TRIP8b_nano_ binding to the CNBD induces a well-defined secondary structure of the distal region of the C-helix (Figure 2). This means that the distal region of the C-helix (residues 657–662), which is unstructured in the free form of the CNBD (Saponaro et al., 2014; Lee and MacKinnon, 2017), extends into a helical structure upon ligand binding irrespectively of whether the ligand is cAMP (Puljung and Zagotta, 2013; Saponaro et al., 2014; Lee and MacKinnon, 2017) or TRIP8b (Figure 2). In contrast, and very differently from cAMP, which directly contacts the P-helix in the Phosphate Binding cassette (PBC) and causes its folding (Saponaro et al., 2014; Lee and MacKinnon, 2017), the NMR data show that TRIP8b_nano_ binding to the CNBD does not induce P-helix formation (Figure 2).

### Modeling the CNBD - TRIP8b_nano_ complex

**Figure 3.**
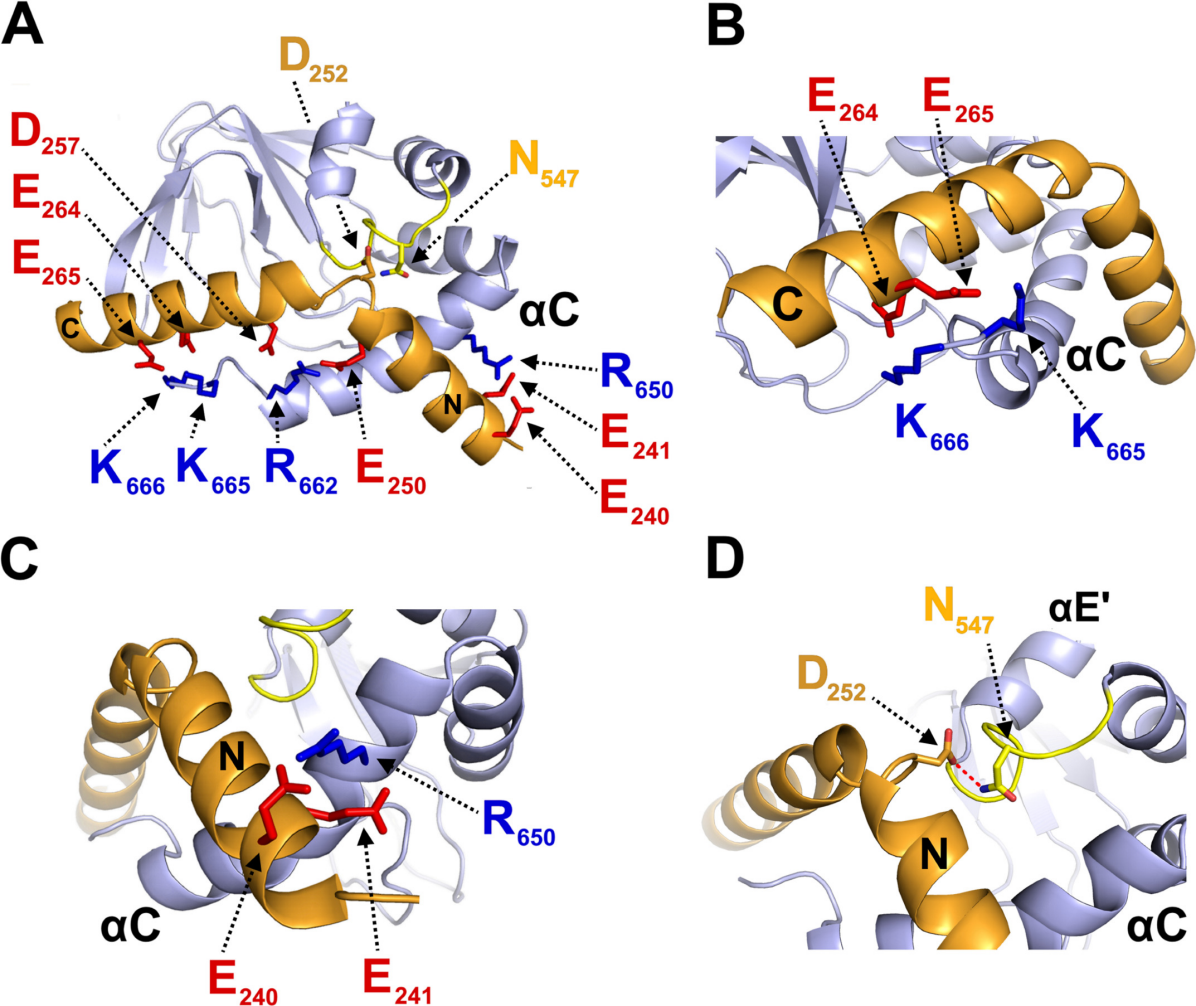
Structural model of CNBD – TRIP8b_nano_ complex. (**A**) Ribbon representation of the complex where CNBD is in gray and TRIP8b_nano_ is in orange. Helix N (**N**) and helix C (**C**) of TRIP8b_nano_ are labeled. C-helix of CNBD (αC) is labeled, while N-bundle loop is colored in yellow. Positively charged residues of C-helix CNBD (blue) and negatively charged residues of TRIP8b_nano_ (red) involved in salt bridges are shown as sticks and labeled. N_547_ of the N-bundle loop (yellow) and D_252_ of TRIP8b_nano_ (orange) are shown as sticks and labeled. (**B**) Close view of K_665_ and K_666_ of CNBD that interact, respectively, with E_265_ and E_264_ of TRIP8b_nano_. C-helix (αC) of CNBD, and Helix C (**C**) of TRIP8b_nano_ are labeled. (**C**) Close view of R_650_ of CNBD that is positioned between E_240_ and E_241_ of TRIP8b_nano_. C-helix (αC) of CNBD, and Helix N (**N**) of TRIP8b_nano_ are labeled. (**D**) Close view of N_547_ of N-bundle loop that forms a hydrogen bond (red dashed line) with D_252_ of TRIP8b_nano_. Helix E’ (αE') and C-helix (αC) of CNBD, and Helix N (**N**) of TRIP8b_nano_ are labeled. 10.7554/eLife.35753.011Figure 3—source data 1.Acquisition parameters for NMR experiments performed on cAMP-free human HCN2 CNBD in complex with TRIP8b_nano_ and vice-versa. 10.7554/eLife.35753.012Figure 3—source data 2.Docking calculation.

Despite the significant improvement in sample stability and NMR spectra quality achieved upon TRIP8b_nano_ binding, we were still unable to assign the side chains of both proteins in the complex and thus could not solve the solution structure of the complex by the canonical NMR procedure. We therefore built a model (Figure 3) of the CNBD - TRIP8b_nano_ complex by docking the two NMR-derived structures described above using the Haddock program (a detailed description of how the respective structures were generated is provided in Materials and methods and Figure 3—source data 1).

**Figure 3—figure supplement 1.**
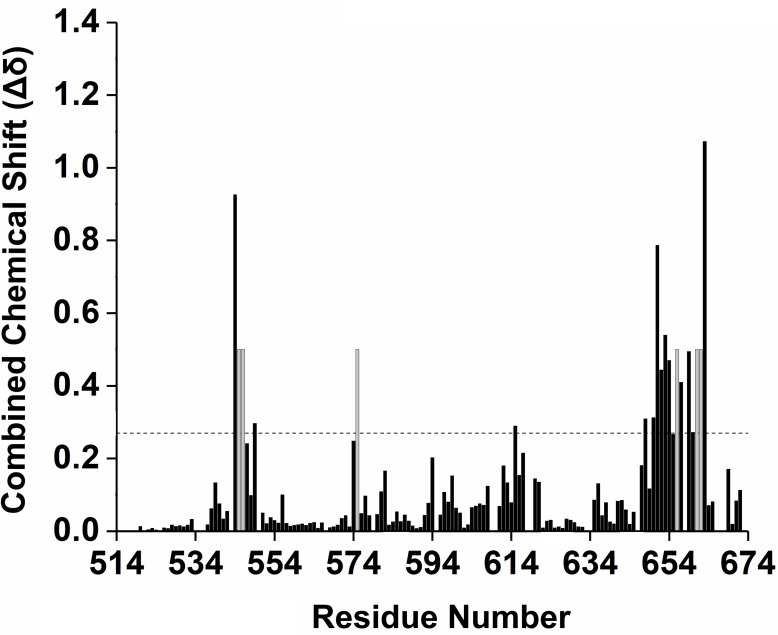
CNBD residues involved in TRIP8b_nano_ binding. Combined chemical shift variations of NMR signals between CNBD unbound and TRIP8b_nano_-bound state. Combined chemical shift variations are calculated from the experimental ^1^H and ^15^N chemical shift changes (Δδ(^1^H) and Δδ (^15^N), respectively) between the corresponding peaks in the two forms, through the following equation (Garrett et al., 1997): ${∆\partial }^{combined}=\sqrt{\frac{{\left(∆\partial \left(1_{H}\right)\right)}^{2}+\frac{1}{25}{\left(∆\partial \left(15N\right)\right)}^{2}}{2}}$. Residues experiencing intermediate exchange regime (whose NMR signal becomes broad beyond detection upon addition of TRIP8b_nano_) are shown in grey. The horizontal dotted line indicates the average value plus one standard deviation. Residues above the line were set as ‘active’ in the docking calculation described in the text (see Materials and methods).

**Figure 3—figure supplement 2.**
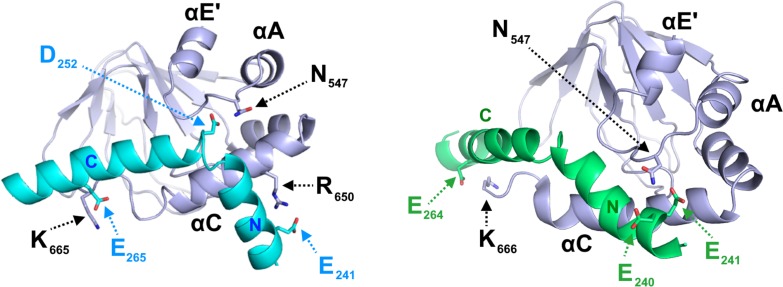
Representative families of clusters obtained from the first docking calculation. The clusters obtained can be grouped in two classes, shown here, on the basis of the position of helix N of TRIP8b_nano_ (N) relative to the CNBD. In the left representative structure, helix N is oriented in a way that allows E_241_ to interact with R_650_ of C-helix. In the right representative structure, E_240_ - E_241_ interact with N_547_ of CNBD. In all clusters K_665_ or K_666_ of CNBD establish a contact with either E_264_ or E_265_ of TRIP8b_nano_. The structural elements of CNBD are labeled: αE’, αA and αC. Helices N and C of TRIP8b_nano_ are labeled.

**Figure 3—figure supplement 3.**
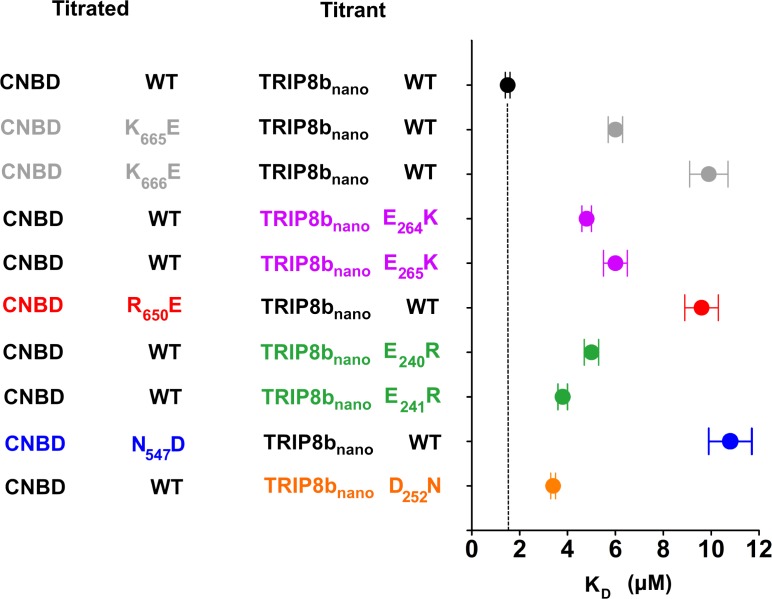
Biochemical validation of CNBD – TRIP8b_nano_ complex. Dissociation constant (K_D_) of the interaction between the indicated CNBD and TRIP8b_nano_ peptides were measured by means of Isothermal Titration Calorimetry (ITC). CNBD WT - TRIP8b_nano_ WT (black filled circle)=1.4 ± 0.1 µM; CNBD K_665_E - TRIP8b_nano_ WT (grey filled circle)=6 ± 0.3 µM; CNBD K_666_E - TRIP8b_nano_ WT (grey filled circle)=9.9 ± 0.8 µM; CNBD WT - TRIP8b_nano_ E_64_K (purple filled circle)=4.8 ± 0.2 µM; CNBD WT - TRIP8b_nano_ E_65_K (purple filled circle)=6 ± 0.5 µM; CNBD R_650_E - TRIP8b_nano_ WT (red filled circle)=9.6 ± 0.7 µM; CNBD WT - TRIP8b_nano_ E_40_R (green filled circle)=5 ± 0.3 µM; CNBD WT - TRIP8b_nano_ E_41_R (green filled circle)=3.8 ± 0.2 µM; CNBD N_547_D - TRIP8b_nano_ WT (blue filled circle)=11 ± 0.9 µM; CNBD WT - TRIP8b_nano_ D_52_N (orange filled circle)=3.4 ± 0.1 µM. Data are presented as mean ± SEM. Number of experiments (**N**) ≥ 3. K_D_ values of all tested combinations are statistically different from the K_D_ of CNBD WT for TRIP8b_nano_ WT (*p≤0.05; **p<0.01). Statistical analysis performed with ANOVA, followed by post-hoc Tukey test. Dashed black vertical line indicates the K_D_ of WT peptides.

**Figure 3—figure supplement 4.**
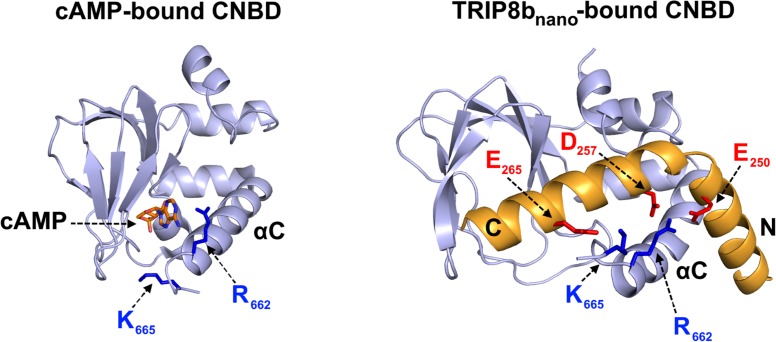
Different orientation of R_662_ and K_665_ in the cAMP-bound and TRIP8b_nano_-bound conformation of the CNBD. Residues R_662_ and K_665_ of CNBD C-helix (αC) which interact with cAMP (left, [Lolicato et al., 2011]) and TRIP8b_nano_ (right, this study) are represented as blue sticks and labeled. TRIP8b_nano_ residues E_250_ and D_257_ interacting with R_662_ and residue E_265_ interacting with K_665_ are shown in red sticks and labeled.

**Figure 3—figure supplement 5.**
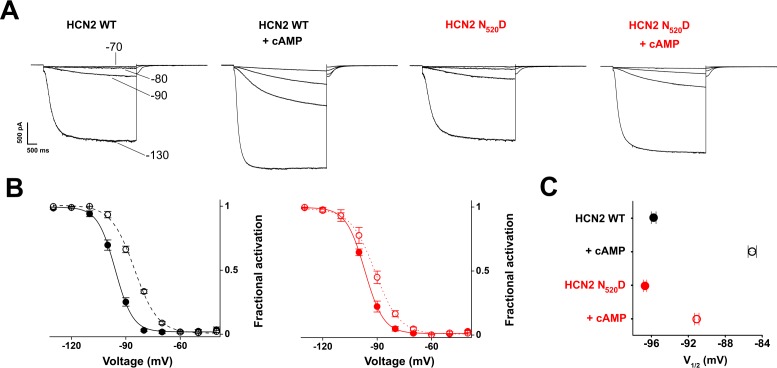
Mutation N_520_D affects cAMP affinity in full-length HCN2 channel. Note that in mouse HCN2 N_520_D corresponds to N_547_D in human HCN2 (used in our structural studies). (**A**) Representative whole-cell HCN2 currents recorded, at the indicated voltages (−70, –80, -90, –130 mV) in HEK 293 T cells transfected with WT and N_520_D mutant, in the absence and presence of 5 µM cAMP in the pipette. (**B**) Mean activation curves obtained from the tail currents in (**A**) and other recordings in control solution (filled circle) and in the presence of cAMP (open circles), with HCN2 wt (black) and N_547_D mutant. Data were fitted to a Boltzmann equation (solid and dotted lines) (see Materials and methods). (**C**) Half activation potential (V_1/2_) values derived from Boltzmann equation fitting. HCN2 WT (black filled circle) V_1/2_ = -95.9 ± 0.3 mV; HCN2 WT + 5 µM cAMP (black open circle) V_1/2_ = 85.2 ± 0.4 mV; HCN2 N_520_D (red filled circle) V_1/2_ = -96.8 ± 0.2 mV; HCN2 N_520_D + 5 µM cAMP (red open circle) V_1/2_ = -91.2 ± 0.2 mV. Data are presented as mean ± SEM. Number of experiments (**N**) ≥ 6. Statistical analysis performed with two-way ANOVA, followed by post-hoc Tukey test (p<0.001) showed that wt and mutant channel are identical and that the cAMP-induced shifts are different.

**Figure 3—figure supplement 6.**
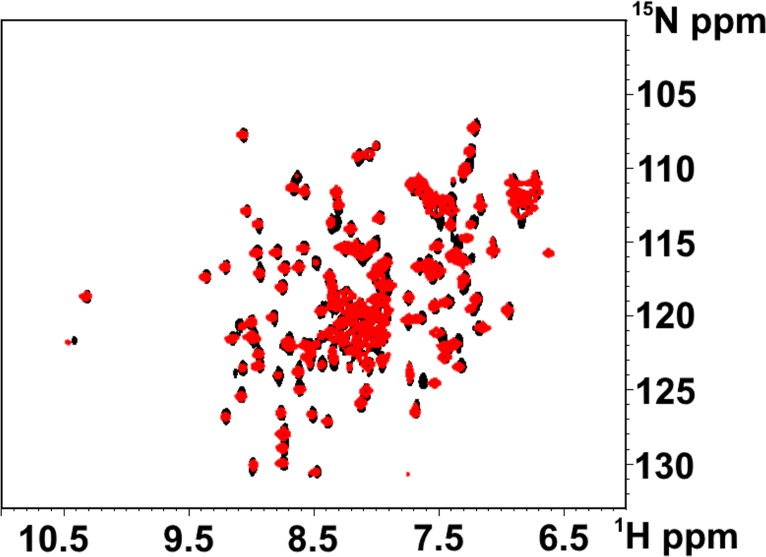
Structural characterization of N_547_D CNBD protein. Superimposition of the [^1^H, ^15^N] HSQC spectra of CNBD wt (black) and CNBD N_547_D mutant (red).

In order to define the active residues (ambiguous interaction restraints) on the CNBD we used the chemical shift perturbation values as described in Figure 3—figure supplement 1. For TRIP8b_nano_, we defined as active a stretch of residues, E_239_-E_243_, previously identified as critical for the interaction (Santoro et al., 2011). Output clusters of this first molecular docking calculation (settings can be found in Materials and methods) were further screened for TRIP8b_nano_ orientations in agreement with a previous DEER analysis, which placed TRIP8b residue A_248_ closer to the proximal portion and TRIP8b residue A_261_ closer to the distal portion of the CNBD C-helix (DeBerg et al., 2015). Remarkably, in all clusters selected in this way, residues E_264_ or E_265_ in TRIP8b were found to interact with residues K_665_ or K_666_ of the CNBD (Figure 3—figure supplement 2). This finding was notable, because we previously identified K_665_/K_666_ as being critical for TRIP8b interaction in a biochemical binding assay (Saponaro et al., 2014). We thus proceeded to individually mutate each of these four positions, and test their effect on binding affinity through ITC. As expected, reverse charge mutations K_665_E or K_666_E (CNBD) as well as E_264_K or E_265_K (TRIP8b_nano_) each strongly reduced the CNBD/TRIP8b_nano_ binding affinity (Figure 3—figure supplement 3).

Based on these observations, we performed a second molecular docking calculation, including E_264_ and E_265_ as additional active residues for TRIP8b_nano_. This procedure resulted in the model shown in Figure 3, which represents the top-ranking cluster for energetic and scoring function (Figure 3—source data 2) and was fully validated by mutagenesis analysis as described below. Scrutiny of the model shows that TRIP8b_nano_ binds to both the C-helix and the N-bundle loop (Figure 3A). Binding to the C-helix is mainly guided by electrostatic interactions between the negative charges on TRIP8b_nano_, and the positive charges on the CNBD (Figure 3A). As shown in Figure 3B, the model highlights a double saline bridge (K_665_ and K_666_ of CNBD with E_265_ and E_264_ of TRIP8b_nano_) in line with the ITC results described above (Figure 3—figure supplement 3). Of note, the contribution of residue R_662_ to the binding is also consistent with previous experiments showing residual TRIP8b interaction in a CNBD deletion mutant ending at position 663 (Saponaro et al., 2014). Our modeling data suggest that, upon folding of the distal portion of the C-helix, the side chains of residues R_662_ and K_665_ face to the inside when contacting cAMP, but face to the outside when binding TRIP8b (Figure 3—figure supplement 4). This indicates that cAMP and TRIP8b directly compete for the binding to the distal region of C-helix.

In addition to clarifying the role of residues in the distal portion of the CNBD C-helix, the model also highlights a second important cluster of electrostatic interactions, with R_650_ in the proximal portion of the CNBD C-helix contacting E_240_ and E_241_ in helix N of TRIP8b_nano_ (Figure 3C). To confirm the contribution of these residues, we reversed charges and tested each residue mutation for binding in ITC. The results in Figure 3—figure supplement 3 show that R_650_E caused a more than six-fold reduction in binding affinity for TRIP8b_nano_, with smaller but significant effects seen also for E_240_R and E_241_R.

A third important contact highlighted by the model is the interaction between N_547_ in the N-bundle loop of the CNBD and D_252_ in the link between helix N and helix C of TRIP8b_nano_ (Figure 3D). We tested this potential interaction by disrupting the expected hydrogen bond between N_547_ and the carboxyl group of the negative residue (D_252_) in TRIP8b_nano_. The asparagine in CNBD was mutated into aspartate (N_547_D) to generate an electrostatic repulsion for D_252_, and the carboxyl group in D_252_ of TRIP8b_nano_ was removed by mutation into asparagine (D_252_N). As predicted, N_547_D greatly reduced binding to TRIP8b in ITC assays (Figure 3—figure supplement 3), with a smaller but significant effect observed also for D_252_N (Figure 3—figure supplement 3). These results confirm and extend our previous finding that the N-bundle loop contributes in a substantial manner to the binding of TRIP8b (Saponaro et al., 2014). To understand the mechanism for the allosteric effect of TRIP8b on cAMP binding, which has been postulated on the basis of electrophysiological and structural data (Hu et al., 2013; Saponaro et al., 2014), we further tested by ITC the affinity of the N_547_D CNBD mutant for cAMP. Somewhat surprisingly, the affinity of the mutant is much lower than that of the wt (N_547_D K_D_ = 5.5 ± 0.4 µM (n = 3) *vs*. wt K_D_ = 1.4 ± 0.1 µM (n = 3)). Moreover, we measured a reduced sensitivity to cAMP also in patch clamp experiments where addition of 5 μM cAMP caused a right shift in the V_1/2_ of the mutant HCN2 channel of only 5 mV while the wt channel shifted by 12 mV (Figure 3—figure supplement 5). To exclude that the N_547_D mutation affects the overall structure of the CNBD, we performed the NMR (^1^H-^15^N HSQC spectrum) analysis of the N_547_D CNBD mutant. Our data show that the protein is appropriately folded (Figure 3—figure supplement 6). In conclusion, since the N-bundle loop does not directly contact any of the residues of the cAMP binding pocket, these findings underscore a previously unaddressed role of the N-bundle loop in allosterically modulating cAMP binding to the CNBD (see Discussion).

### TRIP8b_nano_ as a tool for the direct regulation of native HCN currents

**Figure 4.**
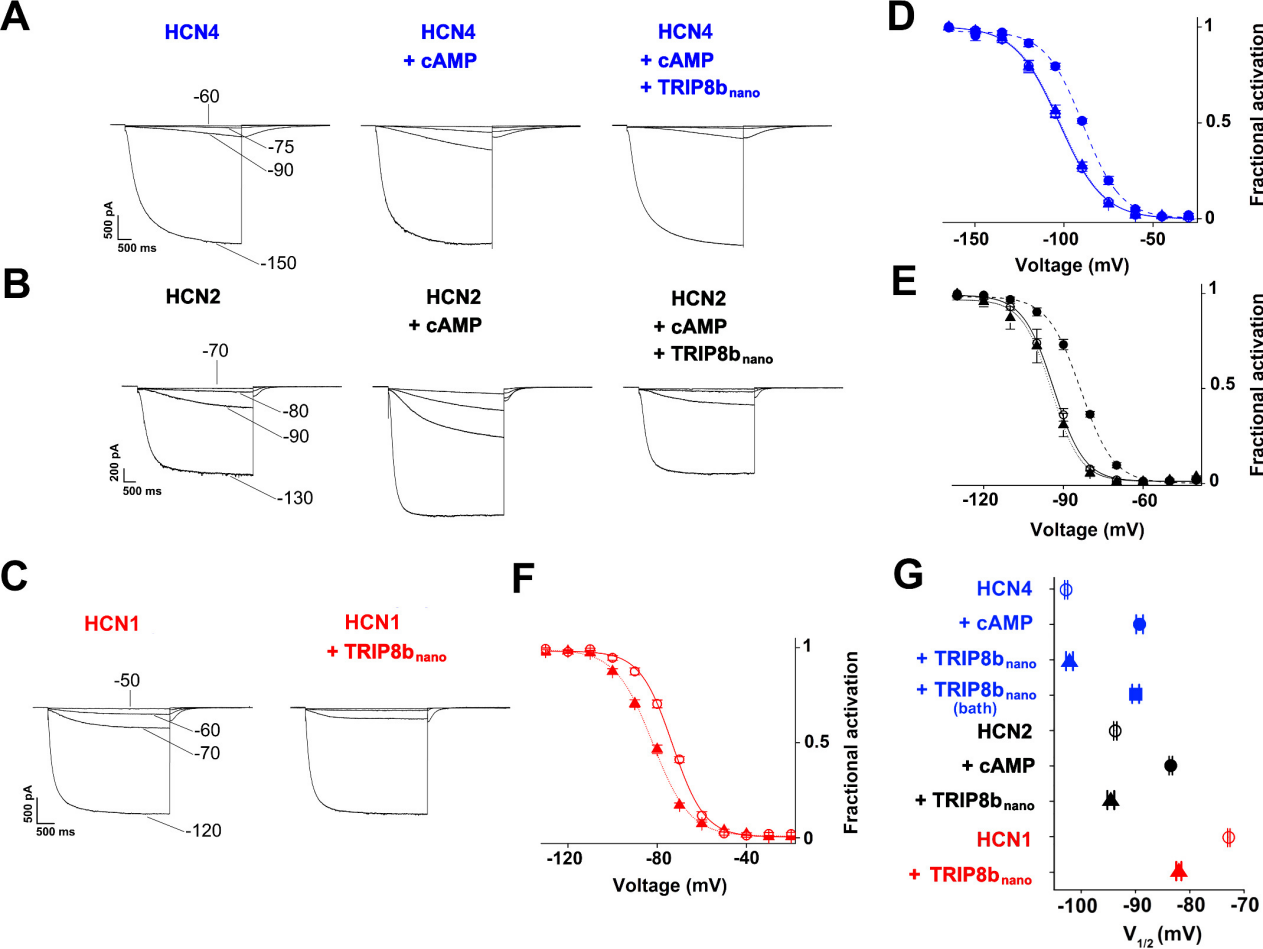
TRIP8b_nano_ abolishes cAMP effect on HCN channel gating. (**A-C**) Representative whole-cell HCN4, HCN2 and HCN1 currents recorded, at the indicated voltages, with control solution or with cAMP (1 μM for HCN4 and 5 μM for HCN2) or with cAMP + 10 µM TRIP8b_nano_ in the patch pipette (for HCN1, 10 µM TRIP8b_nano_ only was added). (**D-F**) Mean activation curves measured from HCN4, HCN2 and HCN1 in control solution (open circles), cAMP (filled circles), cAMP +TRIP8b_nano_, or TRIP8b_nano_ only in the case of HCN1 (filled triangles). Solid and dashed lines indicate Boltzmann fitting to the data (see Materials and methods). (**G**) Half activation potential (V_1/2_) values of HCN4 (blue), HCN2 (black) HCN1 (red) in control solution (open circle), cAMP (filled circle) and cAMP +TRIP8b_nano_, or TRIP8b_nano_ only in the case of HCN1 (filled triangle). HCN4, control = −102.8 ± 0.3 mV; HCN4 +1 µM cAMP = −89.2 ± 0.6 mV; HCN4 +1 µM cAMP +10 µM TRIP8b_nano_ = −102.1 ± 0.6 mV, HCN4 +1 µM cAMP in the patch pipette +10 µM TRIP8b_nano_ in the bath solution = −91.7 ± 0.3 mV; HCN2, control = −93.7 ± 0.3 mV; HCN2 +5 µM cAMP = −83.5 ± 0.3 mV; HCN2 +5 µM cAMP +10 µM TRIP8b_nano_ = −94.5 ± 0.6 mV; HCN1, control = −72.8 ± 0.2 mV; HCN1 +10 µM TRIP8b_nano_ = −82 ± 0.5 mV. Data are presented as mean ± SEM. Number of cells (**N**) ≥ 11. There is no significant difference between the controls and the addition of TRIP8b_nano_ with (HCN4, HCN2) or without (HCN1) cAMP in the pipette. No significant difference was observed following the addition of TRIP8b_nano_ in the bath. Statistical analysis performed with two-way ANOVA, followed by post-hoc Tukey test (p<0.001).

Next, we asked whether the relatively short TRIP8b_nano_ could be used to block cAMP-dependent modulation of HCN channels by delivering the peptide to full length channels. To this end, we dialyzed TRIP8b_nano_ into the cytosol of HEK 293 T cells transfected either with HCN1, HCN2, or HCN4 channels. The peptide was added (10 µM) in the recording pipette together with a non-saturating concentration of cAMP (5 µM for HCN2, 1 µM for HCN4) expected to induce a ~ 10 mV rightward shift in the half-activation potential (V_1/2_) of the channels (Figure 4). No cAMP was added in the case of HCN1, because, in HEK 293 T cells, this isoform is already fully shifted to the right by the endogenous cAMP and does not respond further (Figure 4—figure supplement 1). Indeed, it is possible to induce a ~10 mV left shift in HCN1 V_1/2_ by introducing the mutation R_549_E that prevents cAMP binding to the CNBD (Figure 4—figure supplement 1).

**Figure 4—figure supplement 1.**
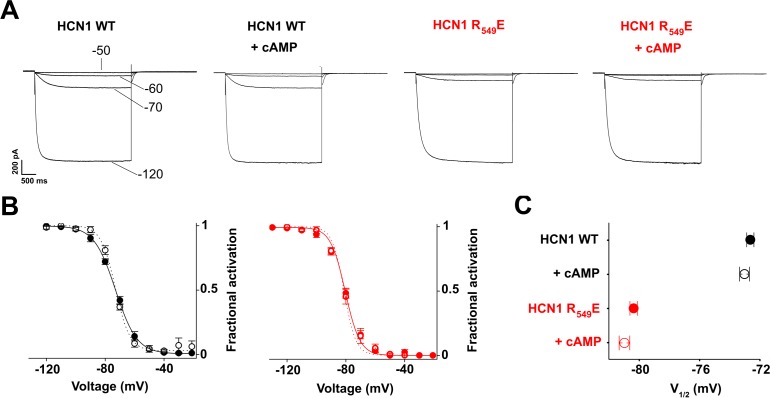
Comparison of half activation potentials (V_1/2_) of HCN1 WT and HCN1 R_549_E mutant (Chen et al., 2001). (**A**) Currents were measured at the indicated voltages (- 50,–60, −70,–120 mV), from HEK 293 T cells with control solution or with 15 μM cAMP in the patch pipette. (**B**) Mean activation curves obtained from tail currents were fitted to a Boltzmann equation (see Materials and methods). (**C**) Half activation potential (V_1/2_) determined from Boltzmann fitting. HCN1 WT (black filled circle)=−72.7 ± 0.2 mV; HCN1 WT + cAMP (black open circle)=−73.1 ± 0.3 mV; HCN1 R_549_E (red filled circle)=−80.4 ± 0.2 mV; HCN1 R_549_E + cAMP (red open circle) = −81 ± 0.3 mV. Note that in human HCN1, used here, R_549_E corresponds to mouse HCN1 R_538_E used in Chen et al. (2001). Data are presented as mean ± SEM. Number of experiments (**N**) ≥ 8. Wt values are significantly different from mutant. Statistical analysis performed with two-way ANOVA, followed by post-hoc Tukey test (p<0.001).

**Figure 4—figure supplement 2.**
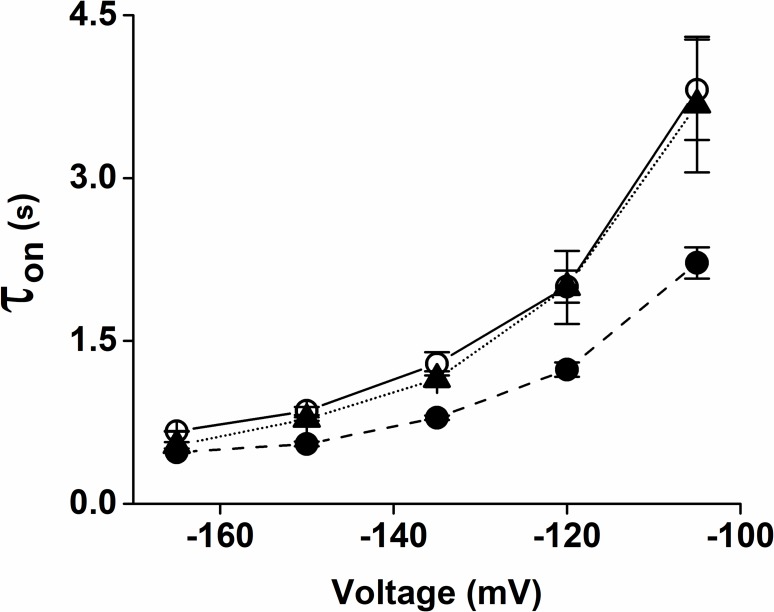
Voltage-dependency of activation time constant (τ_on_) of HCN4 channels in control solution (open circles), 1 µM cAMP (filled circles), 1 µM cAMP + 10 µM TRIP8b_nano_ (filled triangles). Data are presented as mean ± SEM. Number of cells (**N**) = 5.

Figure 4A–C show representative current traces recorded at four given voltages, in control solution, cAMP, and cAMP +10 µM TRIP8b_nano_ (HCN4 and HCN2) or +10 µM TRIP8b_nano _only (HCN1) in the patch pipette. Already from a visual comparison of the most positive voltage at which the current appears measurable, it is evident that TRIP8b_nano_ counteracts the activating effect of cAMP on the voltage-dependent gating. In the case of HCN1, the effect of TRIP8b_nano_ can be observed without added cAMP for the aforementioned reasons. Figure 4D–F show the mean channel activation curves obtained from the above and other experiments. Fitting the Boltzmann equation to the data (solid and dashed lines of Figure 4D–F, see Materials and methods for equation) yielded the half-activation potential values (V_1/2_) plotted in Figure 4G. The addition of TRIP8b_nano_ prevents the cAMP-induced right shift of about 13 mV in HCN4 (V_1/2_ = -102.8, –89.2, −102.1 mV, for control, cAMP and cAMP + TRIP8b_nano_, respectively), and of about 11 mV in HCN2 (V_1/2_ = -93.7, –83.5, −94.5 mV, for control, cAMP and cAMP + TRIP8b_nano_, respectively). In HCN1, TRIP8b_nano_ induced a left shift in V_1/2_ of about 10 mV (from – 72.8 to −82 mV) which is comparable to that induced by the R_549_E mutation (from −72.7 to −80.4 mV) (Figure 4—figure supplement 1).

Figure 4G also shows the result of a control experiment performed on HCN4 where 10 µM TRIP8b_nano_ was added to the extracellular medium (TRIP8b_nano_ bath) in order to test if the peptide was able to cross the cell membrane (current traces and activation curves not shown). The V_1/2_ value, which is similar to that of cAMP alone (−91.7 vs. −89.2 mV), confirmed that TRIP8b_nano_ peptide affects channel gating only if added to the intracellular solution presumably because it does not diffuse through the cell membrane.

It is worth noting that TRIP8b_nano_ prevents other related effects of cAMP activation in HCN channels, such as the acceleration of activation kinetics (Wainger et al., 2001) and, for HCN2 only, the increase in maximal current (Chen et al., 2007; Hu et al., 2013). For example, the activation kinetics (τ_on_) of HCN4 measured at −120 mV was: control = 2 ± 0.2 s, 1 μM cAMP = 1.2 ± 0.1 s, 1 μM cAMP + 10 μM TRIP8b_nano_ = 2 ± 0.3 s (Figure 4—figure supplement 2). Moreover, Figure 4B clearly shows that TRIP8b_nano_ fully prevented the increase in maximal current in HCN2.

**Figure 5.**
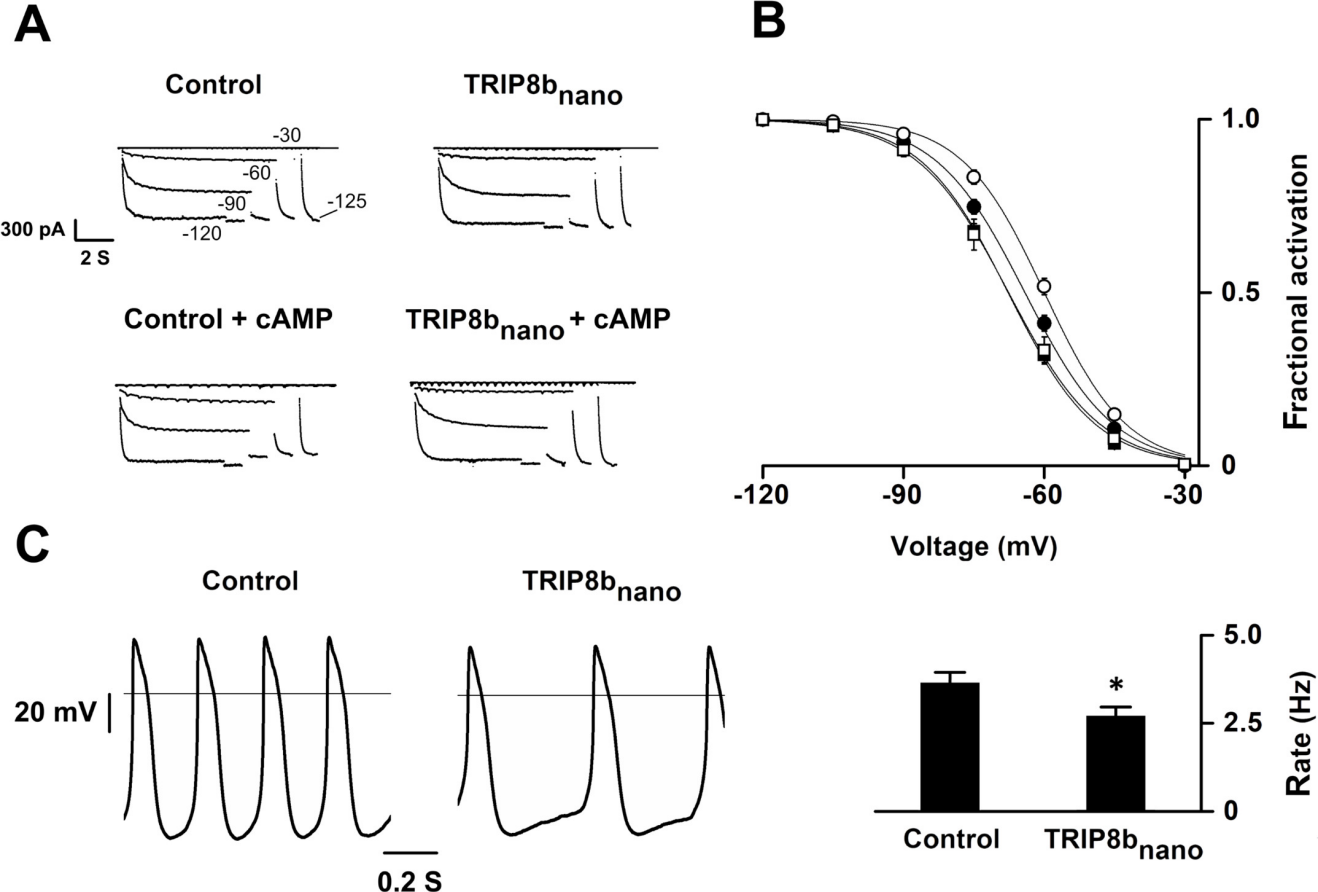
Effects of TRIP8b_nano_ on voltage-dependent activation of I_f_ and spontaneous rate in rabbit sinoatrial node (SAN) myocytes. (**A**) Representative whole-cell I_f_ currents recorded, at the indicated voltages, in the control solution and in the presence of 10 μM TRIP8b_nano_, without (top) and with 1 μM cAMP in the pipette (bottom). (**B**) Mean I_f_ activation curves were measured using a two-step protocol (see Materials and methods) in control (filled circles) or in the presence of: 1 µM cAMP (open circles); 10 µM TRIP8b_nano_ (filled squares); 1 µM cAMP +10 µM TRIP8b_nano_ (open squares). Ligands were added in the patch pipette. Half activation potential (V_1/2_) of I_f_ activation curves measured in control = −64.1 ± 0.4 mV or in the presence of: 1 µM cAMP = −59.9 ± 0.4 mV; 10 µM TRIP8b_nano_ = −67.7 ± 0.4 mV; 1 µM cAMP +10 µM TRIP8b_nano_ = −67.6 ± 0.7 mV. Data are presented as mean ± SEM. Number of cells (**N**) was ≥15. V_1/2_ values are significantly different between each other’s whit the exception of V_1/2_ obtained in the presence TRIP8b_nano_ and cAMP +TRIP8 _bnano_. Statistical analysis performed with two-way ANOVA, followed by post-hoc Bonferroni test (*p<0.05) (**C**) (Left) Representative recordings of single SAN cell spontaneous activity in control and in the presence of 10 µM TRIP8b_nano_. (Right) Mean spontaneous rate (Hz) recorded in control solution = 3.65 ± 0.29 Hz and in the presence of 10 µM TRIP8b_nano_ added to the pipette = 2.69 ± 0.27 Hz. Data are presented as mean ± SEM. Number of cells (**N**) was ≥7. Statistical analysis performed with t test (*p<0.05).

Based on these results, we reckoned the peptide may be employed as a regulatory tool for native I_f_/I_h_ currents. As proof of principle, we tested whether TRIP8b_nano_ can modulate the frequency of action potential firing in SAN myocytes. In these cells, I_f_ is key contributor of the diastolic depolarization phase of the pacemaker action potential cycle. Moreover, the autonomic nervous system modulates the frequency of action potential firing by changing intracellular cAMP levels, which in turn acts on f-HCN channel open probability (DiFrancesco, 1993). We thus recorded the native I_f_ current in acutely isolated rabbit SAN myocytes with and without 10 µM TRIP8b_nano_ in the pipette solution (Figure 5A). Figure 5B shows that the averaged I_f_ activation curve measured in presence of TRIP8b_nano_ is significantly shifted to hyperpolarized voltages compared to the control. This indicates that the peptide is displacing the binding of endogenous cAMP to native HCN channels. Moreover, when the experiment was repeated in the presence of 1 µM cAMP, TRIP8b_nano_ prevented the typical cAMP-dependent potentiation of the native I_f_ current (Figure 5B). In light of these results, we tested whether TRIP8b_nano_ is also able to modulate cardiac automaticity by antagonizing basal cAMP. The data in Figure 5C show that TRIP8b_nano_ indeed significantly decreased the rate of action potential firing in single SAN cells. Strikingly, the observed 30% decrease in action potential rate corresponds to the effect induced by physiological concentrations of acetylcholine (DiFrancesco et al., 1989).

To conclusively prove that the inhibition of the native I_f_ current was specifically due to TRIP8b_nano_ rather than caused by the dilution of the cellular content following whole cell configuration, we created a TAT version of TRIP8b_nano_ (hereafter TAT-TRIP8b_nano_). Indeed, the TAT sequence allows the entry of biomolecules into a cell via endocytosis and/or direct translocation across the plasma membrane, thus leaving the cytosolic content unaltered (Guidotti et al., 2017).

**Figure 6.**
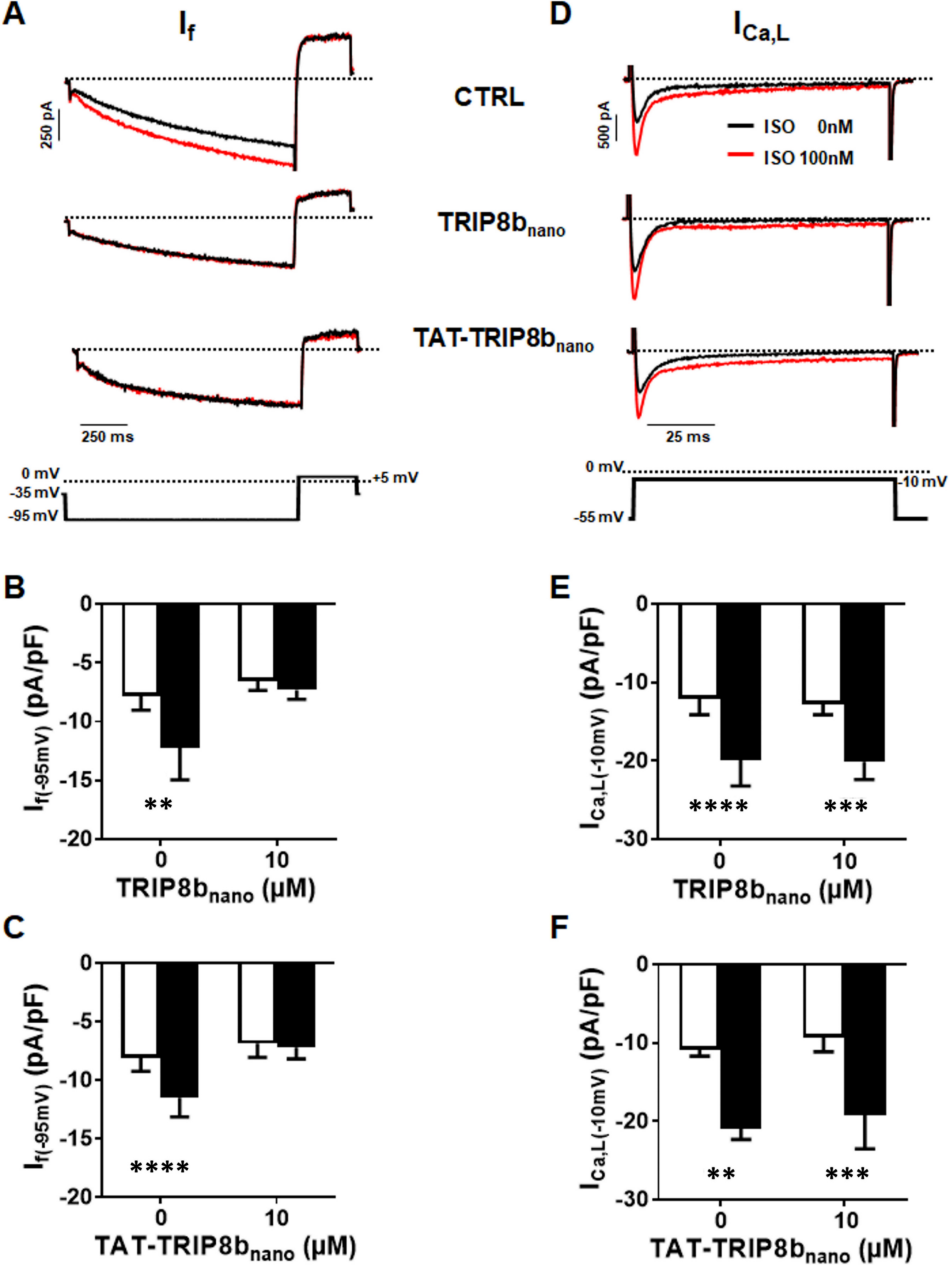
Effect of TRIP8b_nano_ and TAT-TRIP8b_nano_ on I_f_ and I_Ca,L_ in mouse sinoatrial node (SAN) myocytes. (**A**) Representative examples of I_f_ recordings at −95 mV in control conditions (top), in 10 µM TRIP8b_nano_ dialyzed cell (middle) and in cells perfused with 10 µM TAT-TRIP8b_nano_ (bottom), before (black trace) and after (red trace) application of ISO 100 nM. The voltage-clamp protocol used for recordings is shown above current traces. (**B**) Mean normalized I_f_ current density recorded at −95 mV in absence and in presence of 10 µM TRIP8b_nano_ in the patch pipette, before (open bars) and after (filled bars) 100 nM ISO perfusion. Data are presented as mean ± SEM. Number of cells (**N**) ≥ 6. Statistical analysis performed with two-way ANOVA test, followed by Sidak multiple comparisons test (**p<0.01). (**C**) Mean normalised I_f_ current density recorded at −95 mV in control solution or in the solution containing 10 µM TAT-TRIP8b_nano_, in absence (open bars) and in the presence (filled bars) of 100 nM ISO. Data are presented as mean ± SEM. Number of cells (**N**) ≥ 8. Statistical analysis performed with two-way ANOVA test, followed by Sidak multiple comparisons test (****p<0.0001). (**D**) Representative examples of I_Ca,L_ recordings at −10 mV in control conditions (top), in 10 µM TRIP8b_nano_ dialyzed cell (middle) and in cells perfused with 10 µM TAT-TRIP8b_nano_ (bottom), before (black trace) and after (red trace) application of ISO 100 nM. The voltage-clamp protocol used for recordings is shown above current traces. (**E**) Mean normalized I_Ca,L_ current density recorded at −10 mV in absence and in presence of 10 µM TRIP8b_nano_ in the patch pipette, before (open bars) and after (filled bars) 100 nM ISO perfusion. Data are presented as mean ± SEM. Number of cells (**N**) ≥ 8. Statistical analysis performed with two-way ANOVA test, followed by Sidak multiple comparisons test (***p<0.001; ****p<0.0001). (**F**) Mean normalized I_Ca,L_ current density recorded at −10 mV in control solution or in the solution containing 10 µM TAT-TRIP8b_nano_, in absence (open bars) and in the presence (filled bars) of 100 nM ISO. Data are presented as mean ± SEM. Number of cells (**N**) ≥ 7. Statistical analysis performed with two-way ANOVA test, followed by Sidak multiple comparison test (**p<0.01; ***p<0.001).

We therefore tested whether both TRIP8b_nano_ and TAT-TRIP8b_nano_ were able to selectively inhibit the β-adrenergic stimulation of I_f_ current, while leaving the potentiation of L-type Ca^2+^ current (I_Ca,L_) unaltered. To this end, we recorded either the native I_f_ or I_Ca,L_ current from cardiomyocytes acutely isolated from mouse sinoatrial node (SAN) in the presence and in the absence of 10 µM TRIP8b_nano_ or TAT-TRIP8b_nano_, before and after stimulation with 100 nM isoproterenol (ISO), a β-adrenergic receptor agonist (Figure 6). Strikingly, TRIP8b_nano_ prevented the isoproterenol-induced increase of I_f_ current density, both when the peptide was added in the recording pipette solution (Figure 6A and B), and when it was used in the TAT version added to the bath (Figure 6A and C). The specificity of TRIP8b_nano_ for I_f_ current was confirmed by the absent inhibition of basal I_Ca,L_ (Figure 6D). In addition, we failed to record a significant difference in the isoproterenol-stimulated increase of the I_CaL_ current density between the control condition and 10 µM TRIP8b_nano_ (Figure 6E) or TAT-TRIP8b_nano_ (Figure 6F) conditions. To test whether the TAT-TRIP8b_nano_ effect described above was exclusively due to TRIP8b_nano_ peptide, we repeated the experiments with a scrambled version of the peptide (TAT- (SCRAMBLED) TRIP8b_nano_) to exclude that the effect could be due to the TAT sequence (Figure 6—figure supplement 1). We failed to observe a significant reduction in the responsiveness of I_f_ to isoproterenol in the presence of TAT- (SCRAMBLED) TRIP8b_nano_ confirming that prevention of cAMP induced f- current stimulation was specific of the TRIP8b_nano_ sequence.

**Figure 6—figure supplement 1.**
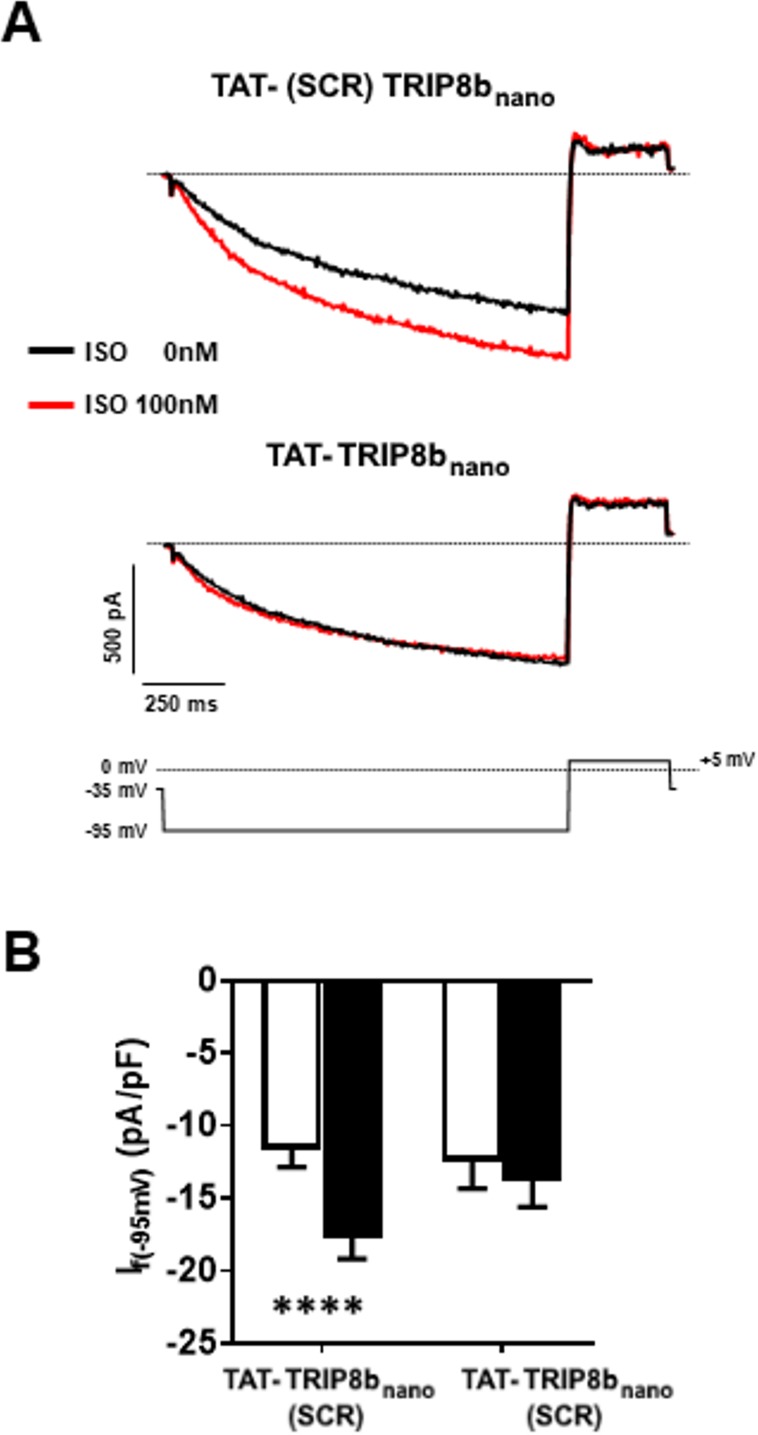
Effect of TAT- (SCRAMBLED) TRIP8b_nano_ on I_f_ in mouse sinoatrial node (SAN) myocytes. (**A**) Representative examples of I_f_ recordings (at −95 mV) in TAT-(SCRAMBLED)TRIP8b_nano_ (top) or in TAT-TRIP8b_nano_ (bottom) incubated cells. The voltage-clamp protocol used for recordings is shown above current traces. (**B**) Mean normalized I_f_ current density recorded at −95 mV in the presence of 10 µM TAT- (SCRAMBLED) TRIP8b_nano_ or TAT-TRIP8b_nano_, before (open bars) and after (filled bars) 100 nM isoprenaline (ISO) perfusion. Data are presented as mean ± SEM. Number of cells (**N**) ≥ 11. Statistical analysis performed with two-way ANOVA test, followed by Sidak multiple comparisons test (****p<0.0001).

## Discussion

### TRIP8b-CNBD complex

In this study, we have identified the minimal binding peptide that reproduces the effects of TRIP8b on HCN channel gating. The peptide is 40 aa long and binds the HCN CNBD with high affinity (K_D_ = 1.4 μM). By solving the NMR structures of TRIP8b_nano_ and HCN CNBD in the bound form, we generated a structural model of their complex. The model provides detailed information on this protein-protein interaction at the atomic level with implications on their physiological function. The data show that the minimal binding unit of TRIP8b, TRIP8b_nano_, folds in two helices upon binding and suggest that this region is intrinsically disordered when it is not bound. The model structurally validates previous indirect evidence, which suggested that TRIP8b binds to two discrete elements of the CNBD: the N-bundle loop and the C-helix (Saponaro et al., 2014). The complex forms by electrostatic interactions, which are spread throughout the contact surface. As a consequence of the interaction with TRIP8b_nano_, the C-helix of CNBD increases in length, a behavior previously observed in the case of cAMP binding (Puljung and Zagotta, 2013). This portion of C-helix includes the two residues R_662_ and K_665_ engaged in salt bridge formation with respectively E_250_/D_257_ and E_264_ of TRIP8b_nano_. It is important to note that these two cationic residues are also involved in cAMP binding (Zagotta et al., 2003; Zhou and Siegelbaum, 2007; Lolicato et al., 2011). The finding that TRIP8b and cAMP share the same binding sites on the C-helix provides a solid molecular explanation for functional data, which imply a competition between the two regulators (Han et al., 2011; DeBerg et al., 2015; Bankston et al., 2017). Another study, however, has indicated that a direct competition model cannot fully explain the mutually antagonistic effect of the two ligands (Hu et al., 2013). Specifically, the fact that the inhibitory effect of TRIP8b on channel activity persists even at saturating cAMP concentrations advocated an allosteric component in the regulation mechanism. Our data, showing that the N_547_D mutation in the N-bundle loop controls cAMP affinity in the binding pocket support the conclusion that the N-bundle loop allosterically controls cAMP binding. This is not surprising, given its crucial role of mechanically transducing to the pore the cAMP binding event within the CNBD (Saponaro et al., 2014).

The structural model also explains why a previously identified peptide selected by Lyman et al. (2017) failed to reproduce the binding affinity of the TRIP8b_core_ for the CNBD. This 37 aa long fragment is lacking one important contact residue, namely E_240, _which, in our model, forms a salt bridge with R_650_ of the CNBD. The loss of one crucial interaction is presumably the reason for the major decrease in affinity (about 20 times lower) reported for this peptide.

### TRIP8b_nano_ as a tool for modulating native I_f_ currents

In functional assays, we showed that TRIP8b_nano_ binds the HCN channel CNBD with high affinity and fully abolishes the cAMP effect in all tested isoforms (HCN1, 2 and 4).

Given the small size of the peptide (<5 kDa), TRIP8b_nano_ is a good candidate for in vivo delivery into intact cells. As a proof of concept, we fused TRIP8b_nano_ to an internalization sequence, the TAT peptide (YGRKKRRQRRRGG). This arginine-rich Cell Penetrating Peptide (CPP) from HIV has been used in several studies as a vehicle for the delivery of large molecules across the plasma membrane (Guidotti et al., 2017). In our case, the challenge was to construct a TAT-fusion protein, which would be efficiently delivered in the cell without compromising TRIP8b_nano_ function. Indeed, covalent conjugation of a CPP may negatively affect both the function of the cargo, and the cell-penetrating efficacy of the CPP-peptide chimera (Kristensen et al., 2016). The design of the construct was greatly supported by the detailed knowledge of the electrostatic interactions with the target protein CNBD, provided by the NMR model structure. This structure suggested that the polycationic TAT sequence would be best linked to the N-terminus of TRIP8b_nano_ to avoid interference with the cationic residues of CNBD, mainly located in the distal region of C-helix, which are crucial for the binding of the peptide. From test experiments with the TAT-TRIP8b_nano_ peptide in SAN myocytes, we can conclude that this strategy was successful in that: (i) the peptide is efficiently delivered inside the cells; (ii) it is kept in its active conformation; (iii) the TAT sequence did not damage cell membranes and did not interfere with the basic features of I_f_ and I_Ca,L_ currents; (iv) the modification did not affect the proteolytic stability of the TRIP8b_nano_ peptide at least in the time frame of our experiments (30 min to 1 hr).

In conclusion, we successfully used the miniaturized TRIP8b_nano_ peptide to selectively control native I_f_ currents and the rate of spontaneous firing in SAN myocytes. Unlike channel blockers, which inhibit ionic currents, the peptide only interferes with the cAMP-based regulation of HCN channels, while leaving basal HCN functions unaltered. In addition and in contrast to even the most selective blockers, it is selective for HCN and it does not interfere with other cAMP-modulated channels present in the SAN, such as L-type Ca^2+^ channels. Collectively, this makes TRIP8b_nano_ a promising tool in targeted therapeutic interventions.

## Materials and methods

**Key resources table:** 

Reagent type species	Designation	Source or reference	Identifiers	Additional information
Gene (human)	HCN1	Xention Ltd. (Cambridge, UK)	NM_021072.3	
Gene (mouse)	HCN2	PMID: 11331358	NM_008226.2	Laboratory of Steven A. Siegelbaum
Gene (rabbit)	HCN4	PMID: 10212270	NM_001082707.1	
Gene (mouse)	TRIP8b	PMID: 19555649		Laboratory of Steven A. Siegelbaum
Strain, strain background (*E. coli*)	DH5α	Thermo Fisher Scientific		
Strain, strain background (*E. coli*)	Stbl2	Thermo Fisher Scientific		
Strain, strain background (*Mus* *musculus*)	Male or female C57BL/6J mice	The Jackson Laboratory	RRID:MGI:5653012	
Strain, strain background (*Oryctolagus* *cuniculus*)	New Zealand white female rabbits	Envigo	ID strain:HsdOkd:NZW	
Cell line (human)	HEK 293T	ATCC	RRID:CVCL_0063	Tested negative for mycoplasma
Biological sample (*Mus musculus*)	Isolated adult Sinoatrial node (SAN) cardiomyocytes	PMID: 11557233		
Biological sample (*Oryctolagus cuniculus*)	Isolated adult Sinoatrial node (SAN) cardiomyocytes	PMID: 2432247		
Recombinant DNA reagent	pET-52b (plasmid)	EMD Millipore		
Recombinant DNA reagent	modified pET-24b (plasmid)	Laboratory of Daniel L. Minor, Jr.		
Recombinant DNA reagent	pcDNA 3.1 (plasmid)	Clontech Laboratories		
Recombinant DNA reagent	pCI (plasmid)	Promega		
Recombinant DNA reagent	TRIP8bnano (cDNA)	This paper		Made by PCR and cloning; see Constructs
Recombinant DNA reagent	TRIP8bnano (E240R) (cDNA)	This paper		Made by site-directed mutagenesis of TRIP8bnano wt; see Constructs
Recombinant DNA reagent	TRIP8bnano (E241R) (cDNA)	This paper		Made by site-directed mutagenesis of TRIP8bnano wt; see Constructs
Recombinant DNA reagent	TRIP8bnano (E264K) (cDNA)	This paper		Made by site-directed mutagenesis of TRIP8bnano wt; see Constructs
Recombinant DNA reagent	TRIP8bnano (E265K) (cDNA)	This paper		Made by site-directed mutagenesis of TRIP8bnano wt; see Constructs
Recombinant DNA reagent	TRIP8bnano (D252N) (cDNA)	This paper		Made by site-directed mutagenesis of TRIP8bnano wt; see Constructs
Recombinant DNA reagent	TRIP8bcore (cDNA)	PMID: 25197093		
Recombinant DNA reagent	human HCN2 CNBD (cDNA)	PMID: 25197093		
Recombinant DNA reagent	human HCN2 CNBD (N547D) (cDNA)	This paper		Made by site-directed mutagenesis of human HCN2 CNBD wt; see Constructs
Recombinant DNA reagent	human HCN2 CNBD (K665E) (cDNA)	This paper		Made by site-directed mutagenesis of human HCN2 CNBD wt; see Constructs
Recombinant DNA reagent	human HCN2 CNBD (K666E) (cDNA)	This paper		Made by site-directed mutagenesis of human HCN2 CNBD wt; see Constructs
Recombinant DNA reagent	human HCN2 CNBD (R650E) (cDNA)	This paper		Made by site-directed mutagenesis of human HCN2 CNBD wt; see Constructs
Recombinant DNA reagent	human HCN1 (cDNA)	Xention Ltd. (Cambridge, UK)		
Recombinant DNA reagent	TRIP8b (1a4) (cDNA)	This paper		Made by PCR and cloning; see Constructs
Recombinant DNA reagent	mouse HCN2 (cDNA)	Laboratory of Steven A. Siegelbaum		
Recombinant DNA reagent	rabbit HCN4 (cDNA)	PMID: 10212270		
Recombinant DNA reagent	mouse HCN2 (N520D) (cDNA)	This paper		Made by site-directed mutagenesis of mouse HCN2 wt; see Constructs
Sequence-based reagent	human HCN1 (R549E) (cDNA)	This paper		Made by site-directed mutagenesis of human HCN1 wt; see Constructs
Peptide, recombinant protein	TAT-TRIP8b_nano_ (YGRKKRRQRRRGG- NHSLEEEFERAKAAVESTEFWDKMQA EWEEMARRNWISEN)	CASLO ApS		
Peptide, recombinant protein	TAT-(SCRAMBLED)TRIP8b_nano_ (YGRKKRRQRRRGG-RNEAEAAE VAQKDMINERARTHEFEWESWE MWENLSESFK)	CASLO ApS		
Commercial assay or kit	QuickChange Lightning Site- Directed Mutagenesis Kit	Agilent		
Commercial assay or kit	Thermo Scientific TurboFect Transfection Reagent	Thermo Fisher Scientific		
Chemical compound, drug	Adenosine 3', 5'-cyclic monophosphate (cAMP)	SIGMA		
Software, algorithm	Clampfit 10.5/10.7	Molecular Devices	RRID:SCR_011323	
Software, algorithm	CYANA-2.1	L. A. Systems, Inc.		
Software, algorithm	AMBER 12.0	http://pyenmr.cerm.unifi.it/ access/index/amps-nmr		
Software, algorithm	HADDOCK2.2	www.wenmr.eu		

### Constructs

The cDNA fragment encoding residues 235–275 (TRIP8b_nano_) of mouse TRIP8b (splice variant 1a4) was cloned into pET-52b (EMD Millipore) downstream of a Strep (II) tag sequence, while the cDNA fragment encoding residues 521–672 of human HCN2 (HCN2 CNBD) was cloned, in a previous study, into a modified pET-24b downstream of a double His_6_-maltose-binding protein (MBP) (Saponaro et al., 2014). The cDNA encoding full-length human HCN1 channel and mouse TRIP8b (1a4) were cloned into the mammalian expression vector pcDNA 3.1 (Clontech Laboratories), while mouse HCN2 channel and rabbit HCN4 channel were cloned into the mammalian expression vector pCI (Promega). Mutations were generated by site-directed mutagenesis (QuikChange site-directed mutagenesis kit; Agilent Technologies) and confirmed by sequencing.

### Preparation of proteins

The HCN2 CNBD WT and mutant proteins, as well as the TRIP8b_core_ and TRIP8b_nano_ proteins (WT and mutants) were produced and purified following the procedure previously described (Saponaro et al., 2014).

### Structure calculation of the cAMP-free human HCN2 CNBD in complex with TRIP8b_nano_ and vice versa

NMR experiments were acquired on Bruker Avance III 950, 700 and 500 MHz NMR spectrometers equipped with a TXI-cryoprobe at 298 K. The acquired triple resonance NMR experiments for the assignment of backbone resonances of cAMP-free HCN2 CNBD (CNBD hereafter) in complex with TRIP8b_nano_ and vice versa are summarized in Figure 3-source data 1. ^15^N, ^13^C’, ^13^C_α_, ^13^C_β_, and H_α_ chemical shifts were used to derive ϕ and ψ dihedral angles by TALOS + program (Cornilescu et al., 1999) for both CNBD and TRIP8b_nano_. For TRIP8b_nano_, CYANA-2.1 structure calculation (Güntert and Buchner, 2015) was performed using 68 ϕ and ψ dihedral angles and 40 backbone hydrogen bonds as input. For CNBD, CYANA-2.1 structure calculation was performed using 108 ϕ and ψ dihedral angles, combined with the NOEs obtained in our previous determination of the cAMP-free form of the CNBD (Saponaro et al., 2014) for those regions not affected by the interaction with TRIP8b_nano_. The 10 conformers of TRIP8b_nano_ and CNBD with the lowest residual target function values were subjected to restrained energy minimization with AMBER 12.0 (Case, 2012) (http://pyenmr.cerm.unifi.it/access/index/amps-nmr) and used as input in docking calculations.

### Docking calculations

Docking calculations were performed with HADDOCK2.2 implemented in the WeNMR/West-Life GRID-enabled web portal (www.wenmr.eu). The docking calculations are driven by ambiguous interaction restraints (AIRs) between all residues involved in the intermolecular interactions (Dominguez et al., 2003). Active residues of the CNBD were defined as the surface exposed residues (at least 50% of solvent accessibility), which show chemical shift perturbation upon TRIP8b_nano_ binding.

The assignment of the CNBD bound to TRIP8b_nano_ allowed to highlight the residues of CNBD whose backbone featured appreciable Combined Chemical Shift Perturbation (CSP) (Figure 3—figure supplement 1). The combined CSP (Δ_HN_) is given by the equation Δ_HN_={((H_Nfree_−H_Nbound_)^2^+((N_free_−N_bound_)/5)^2^)/2}½ (Garrett et al., 1997).

Passive residues of CNBD were defined as the residues close in space to active residues and with at least 50% solvent accessibility.

In the case of TRIP8b_nano_, the conserved stretch E_239_-E_243_, located in helix N, was defined as active region in a first docking calculation, while all the other solvent accessible residues of the peptide were defined as passive. This docking calculation generated several clusters. A post-docking filter step allowed us to select those clusters having an orientation of TRIP8b_nano_ bound to CNBD in agreement with a DEER study on the CNBD - TRIP8b_nano_ interaction (DeBerg et al., 2015). The selected clusters grouped in two classes on the basis of the orientation of helix N of TRIP8b_nano_ (N) relative to CNBD (Figure 3—figure supplement 2. A second docking calculation was subsequently performed introducing also residues E_264_-E_265,_ located in helix C of TRIP8b_nano_ as active residues. The active residues for CNBD were the same used for the first calculation. For this second HADDOCK calculation, 14 clusters were obtained and ranked according to their HADDOCK score. Among them only four clusters showed both an orientation of TRIP8b_nano_ bound to CNBD in agreement with the DEER study (DeBerg et al., 2015) and the involvement of E_239_-E_243_ stretch of TRIP8b_nano_ in the binding to CNBD. These clusters were manually analyzed and subjected to a per-cluster re-analysis following the protocol reported in http://www.bonvinlab.org/software/haddock2.2/analysis/#reanal. From this analysis, it resulted that the top-ranking cluster, i.e. the one with the best energetic and scoring functions, has a conformation in agreement with mutagenesis experiments (Figure 3—figure supplement 3). Energy parameters (van der Waals energy, electrostatic energy, desolvation energy, and the penalty energy due to violation of restraints) for this complex model are reported in Figure 3—source data 2.

Both docking calculations were performed using 10 NMR conformers of both the CNBD and the TRIP8b_nano_ structures calculated as described above. In the TRIP8b_nano_ structures the unfolded N- and C-terminal regions were removed, while in the CNBD structures only the unfolded N-terminal region was removed. This is because the C-terminal region of the CNBD is known to comprise residues involved in TRIP8b_nano_ binding (Saponaro et al., 2014). Flexible regions of the proteins were defined based on the active and passive residues plus two preceding and following residues. The residue solvent accessibility was calculated with the program NACCESS (Hubbard and Hornton, 1993). In the initial rigid body docking calculation phase, 5000 structures of the complex were generated, and the best 400 in terms of total intermolecular energy were further submitted to the semi-flexible simulated annealing and a final refinement in water. Random removal of the restraints was turned off. The number of flexible refinement steps was increased from the default value of 500/500/1000/1000 to 2000/2000/2000/4000. The final 400 structures were then clustered using a cutoff of 5.0 Å of RMSD to take into consideration the smaller size of protein-peptide interface.

### Electrophysiology of HEK 293 T cells

HEK 293 T cells were cultured in Dulbecco’s modified Eagle’s medium (Euroclone) supplemented with 10% fetal bovine serum (Euroclone), 1% Pen Strep (100 U/mL of penicillin and 100 µg/ml of streptomycin), and stored in a 37°C humidified incubator with 5% CO_2_. The plasmid containing cDNA of wild-type and mutant HCN1, HCN2 and HCN4 channels (1 µg) was co-transfected for transient expression into HEK 293 T cells with a plasmid containing cDNA of Green Fluorescent Protein (GFP) (1.3 µg). For co-expression with TRIP8b (1a-4), HEK 293 T cells were transiently transfected with wild-type (wt) and/or mutant human HCN1 cDNA (1 µg), wt TRIP8b (1a-4) cDNA (1 µg) and cDNA of Green Fluorescent Protein (GFP) (0.3 µg).

One day after transfection, GFP-expressing cells were selected for patch-clamp experiments in whole-cell configuration. The experiments were conducted at R.T. The pipette solution in whole cell experiments contained: 10 mM NaCl, 130 mM KCl, 1 mM egtazic acid (EGTA), 0.5 mM MgCl_2_, 2 mM ATP (Mg salt) and 5 mM HEPES–KOH buffer (pH 7.4). The extracellular bath solution contained 110 mM NaCl, 30 mM KCl, 1.8 mM CaCl_2_, 0.5 mM MgCl_2_ and 5 mM HEPES–KOH buffer (pH 7.4).

TRIP8b_nano_ was added (10 µM) to the pipette solution. cAMP was added at different concentration to the pipette solution depending on the HCN isoform used: 0 µM for HCN1, 5 µM for HCN2 and 1 µM for HCN4.

Whole-cell measurements of HCN channels were performed using the following voltage clamp protocol depending on the HCN isoform measured: for HCN1, holding potential was –30 mV (1 s), with steps from –20 mV to –120 mV (10 mV interval, 3.5 s) and tail currents recorded at –40 mV (3 s); for HCN2, holding potential was –30 mV (1 s), with steps from –40 mV to –130 mV (10 mV interval, 5 s) and tail currents recorded at −40 mV (5 s); for HCN4, holding potential was –30 mV (1 s), steps from –30 mV to –165 mV (15 mV interval, 4.5 s) and tail currents were recorded at −40 mV (5 s). Current voltage relations and activation curves were obtained by the above activation and deactivation protocols and analyzed by the Boltzmann equation, see data analysis.

### Isolation and electrophysiology of rabbit sinoatrial node cells

Animal protocols conformed to the guidelines of the care and use of laboratory animals established by Italian and European Directives (D. Lgs n° 2014/26, 2010/63/UE). New Zealand white female rabbits (0.8–1.2 kg) were anesthetized (xylazine 5 mg/Kg, i.m.), and euthanized with an overdose of sodium thiopental (i.v.); hearts were quickly removed, and the SAN region was isolated and cut in small pieces. Single SAN cardiomyocytes were isolated following an enzymatic and mechanical procedure as previously described (DiFrancesco et al., 1986). Following isolation, cells were maintained at 4°C in Tyrode solution: 140 mM NaCl, 5.4 mM KCl, 1.8 mM CaCl_2_, 1 mM MgCl_2_, 5.5 mM D-glucose, 5 mM HEPES-NaOH (pH 7.4).

For patch clamp experiments cells were placed in a chamber on an inverted microscope and experiments were performed in the whole-cell configuration at 35 ± 0.5°C. The pipette solution contained: 10 mM NaCl, 130 mM KCl, 1 mM egtazic acid (EGTA), 0.5 mM MgCl_2_, and 5 mM HEPES–KOH buffer (pH 7.2). The I_f_ current was recorded from single cells superfused with Tyrode solution with 1 mM BaCl_2_, and 2 mM MnCl_2_.

I_f_ activation curves were obtained using a two-step protocol in which test voltage steps (from −30 to −120 mV, 15 mV interval) were applied from a holding potential of −30 mV and were followed by a step to −125 mV. Test steps had variable durations so as to reach steady –state activation at all voltages. Analysis was performed with the Boltzmann equation (see data analysis).

In current-clamp studies, spontaneous action potentials were recorded from single cells superfused with Tyrode solution, and rate was measured from the interval between successive action potential. When indicated cAMP (1 µM) and/or nanoTRIP8b (10 µM) were added to the pipette solution.

### Isolation and electrophysiology of mouse sinoatrial node cells

Mice were killed by cervical dislocation under general anesthesia consisting of 0.01 mg/g xylazine (2% Rompun; Bayer AG), 0.1 mg/g ketamine (Imalgène; Merial) and 0.04 mg/g of Na-pentobarbital (Euthanasol VET, Laboratoire TVM, Lempdes, France), and beating hearts were quickly removed. The SAN region was excised in warmed (35°C) Tyrode’s solution containing: 140 mM NaCl, 5.4 mM KCl, 1.8 mM CaCl_2_, 1 mM MgCl_2_, 1 mM Hepes-NaOH (pH = 7.4), and 5.5 mM D-glucose and cut in strips. Strips were then transferred into a ‘low-Ca^2+^-low-Mg^2+^’ solution containing: 140 mM NaCl; 5.4 mM KCl, 0.5 mM MgCl_2_, 0.2 mM CaCl_2_, 1.2 mM KH_2_PO_4_, 50 mM taurine, 5.5 mM D-glucose, 1 mg/ml bovine serum albumin (BSA), 5 mM Hepes-NaOH (pH = 6.9).

Tissue was digested by adding Liberase TH (0.15 mg/ml, Roche Diagnostics GmbH, Mannheim, Germany), elastase (1.9 U/ml, Worthington, Lakewood). Digestion was carried out for a variable time of 15–18 min at 35°C. Tissue strips were then washed and transferred into a modified ‘Kraftbrühe’ (KB) medium containing: 70 mM L-glutamic acid, 20 mM KCl, 80 mM KOH, (±) 10 mM D- b-OH-butyric acid; 10 mM KH_2_PO_4_, 10 mM taurine, 1 mg/ml BSA and 10 mM Hepes-KOH (pH = 7.4).

Single SAN cells were isolated by manual agitation in KB solution at 35°C for 30–50 s.

Cellular automaticity was recovered by re-adapting the cells to a physiological extracellular Ca^2+^ concentration by addition of a solution containing: 10 mM NaCl, 1.8 mM CaCl_2_ and normal Tyrode solution containing BSA (1 mg/ml). The final storage solution contained: 100 mM NaCl, 35 mM KCl, 1.3 mM CaCl_2_, 0.7 mM MgCl_2_, 14 mM L-glutamic acid, (±) 2 mM D-b-OH-butyric acid, 2 mM KH_2_PO_4_, 2 mM taurine, 1 mg/ml BSA, (pH = 7.4). Cells were then stored at room temperature until use. All chemicals were from SIGMA (St Quentin Fallavier, France).

For electrophysiological recording, SAN cells in the storage solution were harvested in special custom-made recording plexiglas chambers with glass bottoms for proper cell attachment and mounted on the stage of an inverted microscope (Olympus IX71) and perfused with normal Tyrode solution. The recording temperature was 36°C. We used the whole-cell variation of the patch-clamp technique to record cellular ionic currents, by employing a Multiclamp 700B (Axon Instruments Inc., Foster USA) patch clamp amplifier. Recording electrodes were fabricated from borosilicate glass, by employing a WZ DMZ-Universal microelectrode puller (Zeitz-Instruments Vertriebs GmbH, Martinsried, Germany).

I_f_ was recorded under standard whole-cell configuration during perfusion of standard Tyrode’s containing 2 mM BaCl_2_ to block I_K1_. Patch-clamp pipettes were filled with an intracellular solution containing: 130 mM KCl, 10 mM NaCl, 1 mM EGTA, 0.5 mM MgCl_2_ and 5 mM HEPES (pH 7.2).

For recording of L-type Ca^2+^ currents, pipette solution contained: 125 mM CsOH, 20 mM tetraethylammonium chloride (TEA-Cl), 1.2 mM CaCl_2_, 5 mM Mg-ATP, 0.1 mM Li_2_-GTP, 5 mM EGTA and 10 mM HEPES (pH 7.2 with aspartate). 30 µM TTX (Latoxan, Portes lès Valence, France) to block INa was added to external solution containing: 135 mM tetraethylammonium chloride (TEA-Cl), 4 mM CaCl_2_,10 mM 4-amino-pyridine, 1 mM MgCl_2_, 10 mM HEPES and 1 mg/ml Glucose (pH 7.4 with TEA-OH).

Electrodes had a resistance of about 3 MΩ. Seal resistances were in the range of 2–5 GΩ. 10 µM TRIPb8_nano_ was added to pipette solution. 10 µM TAT-TRIPb8_nano_ was added in cell storage solution for at least 30 min before patch clamp recording.

### TAT-peptides

TAT-TRIP8b_nano_ (**YGRKKRRQRRRGG**-NHSLEEEFERAKAAVESTEFWDKMQAEWEEMARRNWISEN, TAT sequence is shown in bold type) and TAT-(SCRAMBLED)TRIP8b_nano_ (**YGRKKRRQRRRGG**-RNEAEAAEVAQKDMINERARTHEFEWESWEMWENLSESFK, TAT sequence is shown in bold type) were purchased from CASLO ApS. TAT-peptides were dissolved in Milliq water (1.5 mM) and added to the petri dish at the final concentration (10 µM) 30 min before current recordings. During the patch clamp experiments, cells were perfused with standard Tyrode with 2 mM BaCl_2_ (see above) without the peptides. Recordings from the same petri dish were performed over a time window of 10 to 60 min in peptide-free solution

### Data analysis

Data were acquired at 1 kHz using an Axopatch 200B amplifier and pClamp10.5 or 10.7 software (Axon Instruments). Data were analyzed off-line using Clampfit 10.5 or 10.7 (Molecular Devices) and Origin 2015 or 16 (OriginLab Corp., Northampton MA). Activation curves were analyzed by the Boltzmann equation, y = 1/{1 + exp[(V−V_1/2_)/s]}, where y is fractional activation, V is voltage, V_1/2_ half-activation voltage, and s the inverse slope factor (mV) (DiFrancesco, 1999). Mean activation curves were obtained by fitting individual curves from each cell to the Boltzmann equation and then averaging all curves obtained. Activation time constants (τ_on_) were obtained by fitting a single exponential function,

I=I_0_ exp(-t/τ) to current traces recorded at the indicated voltages.

### Ethics statement

Experiments on rabbit SAN cells were performed using left-over cells obtained during experiments approved by the Animal Welfare Body of the University of Milan and by the Italian Ministry of Health (license n.1127/2015-PR). Animal procedures were conformed to the guidelines of the care and use of laboratory animals established by Italian and European Directives (D. Lgs n° 2014/26, 2010/63/UE).

Mouse primary pacemaker cells were isolated from adult C57BL/6J mice as previously described (Mangoni and Nargeot, Cardiovasc Res 2001), in accordance with the Guide for the Care and Use of Laboratory Animals (eighth edition, 2011), published by the US National Institute of Health and European directives (2010/63/EU). The protocol was approved by the ethical committee of the University of Montpellier and the French Ministry of Agriculture (protocol N°: 2017010310594939).

## Additional files

Transparent reporting form

### Data availability

All data generated or analysed during this study are included in the manuscript and supporting files. Source data files have been provided for Figure 3.
