# Supplementary material for: A synthetic peptide that prevents cAMP regulation in mammalian hyperpolarization-activated cyclic nucleotide-gated (HCN) channels
Source: eLife. 2018 Jun 20;7:e35753. doi: 10.7554/eLife.35753 (PMC6023613; doi:10.7554/eLife.35753)
Supplement: Figure 3—source data 1. [file elife-35753-fig3-data1.docx]

| Experiments | Dimension of acquired data | | | Spectral width  (ppm) | | | n^a^ |
| --- | --- | --- | --- | --- | --- | --- | --- |
|  | t_1_ t_2_ t_3_ | | | F_1_ F_2_ F_3_ F_3_ | | |  |
| **HCN2 CNBD** ^b^ | | | | | | | |
| ^1^H-^15^N-HSQC | 256(^15^N) | 1024(^1^H) |  | 32 | 16 |  | 8 |
| CBCA(CO)NH | 108(^13^C) | 56(^15^N) | 2048(^1^H) | 72 | 32 | 16 | 16 |
| HNCACB | 108(^13^C) | 56(^15^N) | 2048(^1^H) | 72 | 32 | 16 | 16 |
| HNCO | 80(^13^C) | 56(^15^N) | 2048(^1^H) | 18 | 32 | 16 | 4 |
| HN(CA)CO | 80(^13^C) | 56(^15^N) | 2048(^1^H) | 18 | 32 | 16 | 16 |
| ^15^N-edited [^1^H-^1^H]-NOESY^c^ | 192(^1^H) | 74(^15^N) | 2048(^1^H) | 14 | 70 | 14 | 16 |
| **TRIP8bnano**^d^ | | | | | | | |
| ^1^H-^15^N-HSQC | 128(^15^N) | 1024(^1^H) |  | 40 | 14 |  | 16 |
| CBCA(CO)NH | 88(^13^C) | 48(^15^N) | 2048(^1^H) | 80 | 40 | 16 | 24 |
| HNCACB | 88(^13^C) | 48(^15^N) | 2048(^1^H) | 80 | 40 | 16 | 24 |
| HNCA | 88(^13^C) | 48(^15^N) | 1024(^1^H) | 50 | 40 | 16 | 24 |
| HN(CO)CA | 88(^13^C) | 48(^15^N) | 1024(^1^H) | 50 | 40 | 16 | 24 |

^a^ number of acquired scans. ^b^ Experiments were acquired on a 700 MHz Bruker spectrometer equipped with a triple resonance cryoprobe at 298 K. All the triple resonance (TCI 5-mm) probes used were equipped with Pulsed Field Gradients along the z-axis.

^c 15^N-edited edited 3D NOESY-HSQC experiments was acquired with a mixing time value of 100ms at the Bruker Avance 950 MHz spectrometer. ^d^ Experiments were acquired on a 500 MHz Bruker spectrometer equipped with a triple resonance cryoprobe at 298 K. All 3D and 2D spectra were processed using the standard Bruker software TOPSPIN 2.1 and analyzed through CARA (Keller R *et al*, 2002; Keller RLJ. 2004).

Keller R, Wüthrich KA. (2002) New Software for the Analysis of Protein NMR Spectra.

Keller RLJ. (2004) The Computer Aided Resonance Assignment tutorial. Goldau: CANTINA Verlag.
