# Supplementary material for: A synthetic peptide that prevents cAMP regulation in mammalian hyperpolarization-activated cyclic nucleotide-gated (HCN) channels
Source: eLife. 2018 Jun 20;7:e35753. doi: 10.7554/eLife.35753 (PMC6023613; doi:10.7554/eLife.35753)
Supplement: Figure 3—source data 2. [file elife-35753-fig3-data2.docx]

| **HADDOCK Score ^b^** | -130 (7) |
| --- | --- |
| **RMSD (Å) ^c^** | 1.8 (0.9) |
| **Number of structures** | 71 |
| **BSA (Å^2^) ^d^** | 1968 (106) |
| **EAIR ^e^** | 30.5 (15.2) |
| **Einter^f^** | -398(50) |
| **Enb^g^** | -431(46) |

Statistics on the top-ranking cluster of the structural models of CNBD and TRIP8b_nano_ complex obtained through HADDOCK2.2 calculations and in agreement with previous experimental data (Deberg *et al*, 2015). Averages (standard deviations are reported in parenthesis) were calculated over the best ten structures.

^a^ Cluster rank according to the HADDOCK score. ^b^ HADDOCK score defined as a weighted sum of different energetic terms, such as: van der Waals energy, electrostatic energy, distance restraints energy, buried surface area, binding energy and desolvation energy. ^c^ Backbone RMSD from the lowest HADDOCK score structure in each cluster. Some individual energy terms are also reported: ^d^ Buried surface area, ^e^ distance restraints energy, ^f^ binding energy, ^g^ non-bonded interaction energy.

Deberg HA, Bankston JR, Rosenbaum JC, Brzovic PS, Zagotta WN & Stoll S (2015) Structural mechanism for the regulation of HCN ion channels by the accessory protein TRIP8b. *Structure* **23:** 734–744. Retrieved from https://doi.org/10.1016/j.str.2015.02.007.
